# The *Arctic AβPP* mutation leads to Alzheimer’s disease pathology with highly variable topographic deposition of differentially truncated Aβ

**DOI:** 10.1186/2051-5960-1-60

**Published:** 2013-09-10

**Authors:** Hannu Kalimo, Maciej Lalowski, Nenad Bogdanovic, Ola Philipson, Thomas D Bird, David Nochlin, Gerard D Schellenberg, RoseMarie Brundin, Tommie Olofsson, Rabah Soliymani, Marc Baumann, Oliver Wirths, Thomas A Bayer, Lars NG Nilsson, Hans Basun, Lars Lannfelt, Martin Ingelsson

**Affiliations:** 1 Department of Public Health/Geriatrics, Uppsala University Hospital, Uppsala University, Box 609, SE-751 25, Uppsala, Sweden; 2 Department of Pathology, University and University Hospital of Helsinki, Helsinki, Finland; 3 Meilahti Clinical Proteomics Core Facility and Institute of Biomedicine, Biochemistry and Developmental Biology, Biomedicum Helsinki, University of Helsinki, Helsinki, Finland; 4 Division of Clinical Geriatrics, Department for Neurobiology, Care Sciences and Society, Karolinska Institutet and Karolinska University Hospital, Stockholm, Sweden; 5 Department of Forensic Medicine, Institute of Biomedicine, University of Turku, Turku, Finland; 6 Departments of Neurology and Medicine, and Neuropathology Laboratory, University of Washington School of Medicine and VA Geriatrics Research Center, Seattle, WA, USA; 7 New Jersey Neuroscience Institute at JFK Medical Center, Edison, NJ, USA; 8 Department of Pathology and Laboratory Medicine, Perelman School of Medicine, University of Pennsylvania, Philadelphia, PA, USA; 9 National Board of Forensic Medicine, Department of Forensic Medicine, Uppsala, Sweden; 10 Department of Psychiatry, Division of Molecular Psychiatry, University Medicine Göttingen, Georg-August-University, Göttingen, Germany; 11 Department of Pharmacology, University of Oslo and Oslo University Hospital, Oslo, Norway

**Keywords:** Familial Alzheimer’s disease, Arctic *AβPP* mutation, β-amyloid peptide, Mass spectrometry, Truncation of Aβ, Topography of Aβ, Hyperphosphorylated tau, Neuronal damage

## Abstract

**Background:**

The Arctic mutation (p.E693G/p.E22G)fs within the β-amyloid (Aβ) region of the β-amyloid precursor protein gene causes an autosomal dominant disease with clinical picture of typical Alzheimer’s disease. Here we report the special character of Arctic AD neuropathology in four deceased patients.

**Results:**

Aβ deposition in the brains was wide-spread (Thal phase 5) and profuse. Virtually all parenchymal deposits were composed of non-fibrillar, Congo red negative Aβ aggregates. Congo red only stained angiopathic vessels. Mass spectrometric analyses showed that Aβ deposits contained variably truncated and modified wild type and mutated Aβ species. In three of four Arctic AD brains, most cerebral cortical plaques appeared targetoid with centres containing C-terminally (beyond aa 40) and variably N-terminally truncated Aβ surrounded by coronas immunopositive for Aβ_x-42_. In the fourth patient plaque centres contained almost no Aβ making the plaques ring-shaped. The architectural pattern of plaques also varied between different anatomic regions. Tau pathology corresponded to Braak stage VI, and appeared mainly as delicate neuropil threads (NT) enriched within Aβ plaques. Dystrophic neurites were scarce, while neurofibrillary tangles were relatively common. Neuronal perikarya within the Aβ plaques appeared relatively intact.

**Conclusions:**

In Arctic AD brain differentially truncated abundant Aβ is deposited in plaques of variable numbers and shapes in different regions of the brain (including exceptional targetoid plaques in neocortex). The extracellular non-fibrillar Aβ does not seem to cause overt damage to adjacent neurons or to induce formation of neurofibrillary tangles, supporting the view that intracellular Aβ oligomers are more neurotoxic than extracellular Aβ deposits. However, the enrichment of NTs within plaques suggests some degree of intra-plaque axonal damage including accumulation of hp-tau, which may impair axoplasmic transport, and thereby contribute to synaptic loss. Finally, similarly as the cotton wool plaques in AD resulting from exon 9 deletion in the presenilin-1 gene, the Arctic plaques induced only modest glial and inflammatory tissue reaction.

## Background

Deposition of amyloid-β (Aβ) peptides and hyperphosphorylated tau (hp-tau) as neurofibrillary tangles (NFT), dystrophic neurites (DN) and neuropil threads (NT) are invariably found in the brains of patients with both sporadic and familial forms of Alzheimer’s disease (AD). The majority of the pathogenic mutations in the amyloid-β precursor protein (*AβPP*) gene (http://www.alzforum.org/res/com/mut/app/default.asp) results in either an increase in total Aβ or Aβ42/Aβ40-ratio and often in aggressive plaque pathology. The recently reported protective effect against AD of the p.A673T substitution in AβPP (p.A2T in Aβ peptide) further suggests that Aβ is pivotal for the disease development [1].

Distinct pathological features are seen in *AβPP* mutation carriers as well as in other early-onset familial forms of AD. For example, brains from carriers of mutations in exons 8 and 9 of the presenilin 1 gene (e.g. *PS1*Δ9*)* harbour cotton wool plaques; large ball-like plaques without an amyloid core [2-4].

Similarly, there is a marked phenotypic variation also among patients with various *AβPP* mutations [5], which is exemplified by different substitutions in *AβPP* codon 693. For example, both the Dutch *AβPP* mutation (p.E693Q; reviewed in [6]) and the Italian mutation (p.E693K; [7,8]) within the Aβ sequence cause amyloid angiopathy with intracerebral hemorrhages, whereas parenchymal AD pathology and dementia are subsidiary. Moreover, the Osaka *AβPP* deletion mutation (p.E693Δ) identified in a Japanese pedigree also causes a primarily dementing disease [9]. The *Arctic AβPP* mutation (p.E693G; in Aβ-peptide p.E22G) was initially reported as a polymorphism of unclear pathogenic significance [10]. Subsequently, the same mutation was found to segregate with AD in a Swedish family [11]. Interestingly, the *Arctic AβPP* mutation increased the formation of large soluble Aβ oligomers/protofibrils [11,12], while symptomatic carriers showed low CSF-Aβ42 levels but remained PIB-PET negative [13]. The Osaka *AβPP* deletion mutation (p.E693Δ) also accelerated Aβ oligomerization [9] but it did not cause deposition of fibrillar Aβ *in vivo*, neither in transgenic mice nor in AD patients [9,14].

Elevated intra- or extracellular levels of Aβ oligomers/protofibrils are believed to be of pathogenic significance and neurotoxic effects have been demonstrated both on cultured cells and *in vivo.* For example, intrathecal administration of Aβ oligomers in rats caused impaired learning [15] and extracellular accumulation of soluble dodecameric Aβ in the brains of *AβPP* transgenic mice impaired cognition independently of plaques or neuronal loss [16].

We have previously reported epidemiological and clinical as well as a limited description (based on two Arctic AD brains) of some neuropathological features resulting from the p.E693G mutation in AβPP [17]. Apart from concluding that the clinical features of this mutation are compatible with AD, we identified ring-formed Aβ plaques but did not further study the overall neuropathology. In another, more recent, biochemically oriented study [18] we analysed the composition of Arctic Aβ plaques in the frontal and temporal cortex of two Arctic AD patients (patients Sw1 and Sw2 of this study) using biochemistry (including Aβ ELISA and MALDI-TOF and MALDI-imaging mass spectrometry) and complementary immunohistochemistry and electron microscopy. Aβ ELISA and mass spectrometry analyses on brain cortex samples from one of our patients (patient Sw2 of this study) with the *Arctic AβPP* mutation revealed deposition of a heterogeneous mixture of Aβ peptides with a significant contribution of N-truncated and N-terminally modified Aβ. Moreover, by applying mid-domain, N- and C-terminal specific Aβ antibodies we demonstrated in the temporal cortex (patient Sw2 of this study), the presence of targetoid plaques composed of both N- and C-terminally truncated Aβ [18].

Here, we have extended and made a comprehensive analyses of the neuropathology in *Arctic AβPP* mutation carriers, based on four autopsied brains. We have applied nine well-characterized antibodies to different epitopes spanning the Aβ molecule, including an antibody specific to the Arctic mutation p.E22G and two antibodies recognizing posttranslational modifications of glutamates 3 and 11 of Aβ (cyclization into pyroglutamate). Thus, we could define the pattern of differentially truncated and N-terminally modified Aβ deposition in different regions of the Arctic AD brains, and also correlate these to mass spectroscopic findings in cerebral cortex. In addition, we describe local effects of Aβ on neurons, the association of Aβ with other pathological features, such as accumulation of hp-tau, macro- and microglial reactions, and basic vascular alterations.

## Methods

### Patients and brain specimens

**Figure 1:**
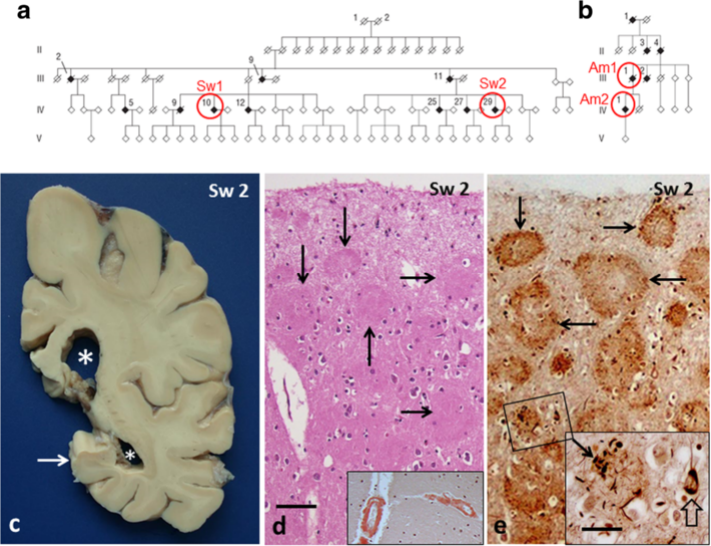
**Pedigrees of the Arctic AD families (a and b) and basic pathology in Sw2 patient (c-e). a** and **b**: Pedigrees of the Swedish and American families with the Arctic *AβPP* gene mutation. Diagonal lines indicate deceased individuals; filled symbols indicate affected and open symbols unaffected members. The patients examined in this study in Family **a** are: IV:10 = Sw1, IV:29 = Sw2 and in Family **b**: III:1 = Am1, IV:1 = Am2. **c**: Sw2 patient’s brain weighed 1490 g. The gyri are only mildly atrophic, whereas the atrophy of hippocampus (arrow) and the dilatation of the ventricular system (asterisks) are obvious. **d** and **e**: Semi-consecutive sections from Sw2 patient’s frontal cortex: **d**: The plaques (five marked with an arrow) are discernible already with H&E staining. They are rounded, compact, eosinophilic structures with homogeneous texture, reminiscent of so called cotton wool plaques [3]. Inset in **d**: Both pial and penetrating arteries are strongly Congo positive, but no plaques are visible. **e**: In a Bielschowsky silver impregnated section several plaques are vaguely ring formed (four marked with an arrow). No prominent dystrophic neurites are seen, only short, thin stubs. Inset (the square in **e**): Occasional plaques harbour somewhat coarser dystrophic neurites (arrow). Open arrow points to a neuron with neurofibrillary tangle. (*bar* in **d** 100 μm for **d** and **e**; *bar* in inset of **e** 40 μm).

Two patients from a Swedish and two from an American family were included in the study. The presence of the Arctic mutation was verified by sequencing of exon 17 of the *AβPP* gene according to the methods previously described [11]. Although not proven, these two families are believed to descend from the same Swedish ancestor, (for respective pedigrees, see Figure 1a-b). The Swedish patients died at 62 (Sw1 = IV:10) and 64 (Sw2 = IV:29) years of age, after six and eight years of disease duration, respectively. The American patients died at 72 (Am1 = III:1) and 59 (Am2 = IV:1) years of age after 16 and 8 years of disease duration, respectively.

This study has been carried out in compliance with the Helsinki Declaration and it has been approved by the Regional ethical committee in Uppsala, Sweden (2009)/089; 2009-04-22 and 2005-103; 2005-2006-29.

The brains were routinely fixed in buffered 4% formaldehyde, within twelve hours *post mortem* and widely sampled for embedding in paraffin. For comparison we used brain samples from five AD patients with the cotton wool plaque-associated *PS1Δ9* mutation [3].

### Histopathology and immunohistochemistry

The basic histopathology was examined in sections stained with hematoxylin and eosin (H&E), Bielschowsky’s silver impregnation, thioflavin-S, Congo red or periodic acid Schiff (PAS) stains. The antibodies used for immunohistochemistry of Aβ hp-tau, astrocytes, microglia and neurons are listed in Table 1. Well characterized antibodies to different epitopes in wild type (wt) Aβ, called *general* Aβ-antibodies (Table 1), were selected to identify epitopes in N-terminal, mid-domain and C-terminal regions of non-mutated Aβ. Among those none is selective for wild-type Aβ and only antibody abAβ_17-24_ (clone 4G8) has reduced affinity for Arctic Aβ. In addition, *specific* antibodies recognizing Aβ with the Arctic mutation and certain post-translational truncations and modifications, were applied (Table 1).

**Table 1:** The list of antibodies used in immunohistochemistry and immunoprecipitation experiments

***Antibody***	***Epitope/Target***	***Manufacturer***
*N-terminal Aβ-antibodies*		
mAb 82E1	Neoepitope Aβ1-5	IBL, Hamburg, Germany
mAb 6E10	Aβ5-10	Covance, Berkeley, CA, USA
*Mid-domain Aβ-antibodies*		
mAb 6F/3D	Aβ8-17	Novocastra, Newcastle, U.K.
mAb 4G8	Aβ17-24	Covance
*C-terminal Aβ-antibodies*		
rpAb 40	Aβx-40, neoepitope	Biosource/Invitrogen, Camarillo CA, USA
rpAb 42	Aβx-42, neoepitope	Biosource/Invitrogen
*Special Aβ-antibodies*		
mAb 27	Aβ20-24 with Arc-mutation p.E22G	Lord et al. 2009 [19]
mAb 2-48	Aβ3pE	Synaptic Systems, Göttingen, Germany
rpAb 11pE	Aβ11pE	Synaptic Systems
*Antibodies to cellular alterations*		
mAb AT8	Hyperphosphorylated tau	Innogenetics, Zwijndrecht, Belgium
mAb GFAP	Glial fibrillary acidic protein	Dako, Glostrup, Denmark
mAb HLA-DP, DQ, DR	Microglial cells	Dako
rpAb Iba1	Microglial cells	Wako, Osaka, Japan
rpAbα1-antitrypsin	Lysosomes	Dako
rpAb cathepsin D	Lysosomes	Dako (production discontinued)

*Abbreviations: mAb* mouse monoclonal antibody, *rpAb* rabbit polyclonal antibody.

After pre-treatment relevant for each antigen, the sections were incubated with the primary antibodies overnight at +4°C, followed by incubation with relevant secondary antibodies and visualization using the avidin-biotin-peroxidase method with diaminobenzidine as chromogen (Vectastain, Vector Laboratories, Burlingame, CA, USA). Neurons and their axons were double-labelled with a polyclonal Aβ_x-40_ antibody and a monoclonal antibody to neurofilament, followed by Alexa 633 labeled anti-rabbit and Alexa 488 labeled anti-mouse secondary antibodies (Molecular Probes, Eugene, OR, USA). The details of antibody sources and specifications are listed in the Table 1.

### Mass spectroscopy

For comparative immunohistochemical and mass spectroscopic analyses of Aβ in cortical plaques, samples of fresh frozen temporal cortex from patient Sw2 were immunoprecipitated (with antibodies to Aβ_17–24_ and Aβ_arc_) and analysed by MALDI-TOF, as described in our previous study [18].

## Results

### Macroscopic pathology

Patient Sw1 (IV:10): ApoE genotype 3/3. The brain weighed 1385 g. Gross examination revealed focal moderate atrophy of parietal superior lobules. There were no signs of infarcts or hemorrhages.

Patient Sw2 (IV:29): ApoE genotype 3/3. The brain weighed 1490 g. A mild degree of atrophy with dilatation of the ventricular system was seen in frontal, parietal and occipital lobes as well as in different parts of the temporal lobe, including gyrus parahippocampalis, hippocampus, and amygdala (Figure 1c). Brainstem and cerebellum had normal macroscopic appearance, apart from mild atrophy of the anterior part of vermis.

Patient Am1 (III:1): ApoE genotype 2/3. The weight of the brain was 822 g. There was a severe degree of atrophy in frontal, temporal and parietal cortices [17].

Patient Am2 (IV:1): ApoE genotype 2/3. After fixation, the brain weighed 1220 g and showed moderate cortical atrophy.

### Microscopic pathology of Aβ deposition

The deposition of Aβ in human brain has been suggested to follow a distinct hierarchical sequence, classified as phases 1 to 5 [20]. In the following, we present the structural and immunohistochemical findings in the anatomical regions corresponding to these phases. The pattern of Aβ deposits varied both with respect to the antibodies applied and to the different brain regions analysed. Furthermore, there were some noticeable differences between the four Arctic AD brains studied.

### Cerebral neocortices (phase 1)

#### Histopathology

In H&E stained sections, senile plaques appeared as compact rounded structures with remarkably homogenous texture (Figure 1d). The plaques were devoid of an amyloid core, as shown by the absence of Congo red (Figure 1d, inset) and thioflavin S (not shown) positivity and thus resembled cotton wool plaques (Additional file 1: Figure S9) [2,3]. With Bielschowsky silver impregnation (without gold enhancement) the plaques were moderately brownish with accentuation of peripheral parts and negative or weakly stained centres, giving the plaques a vaguely ring-like pattern (Figure 1e).

### Aβ immunohistochemistry

#### General features

**Figure 2:**
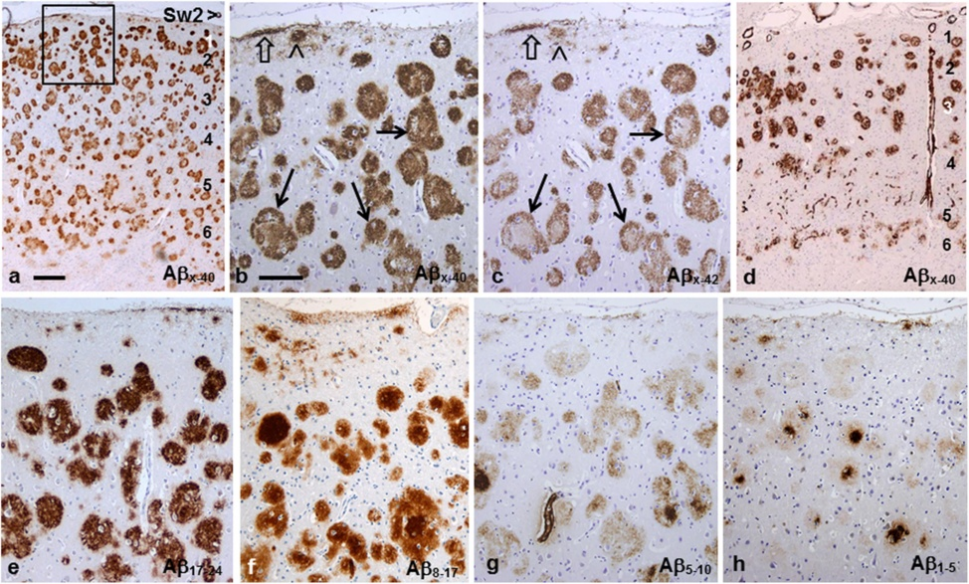
**Aβ immunostainings of Sw2 patient’s frontal cortex. a**: The plaques in frontal cortex are most numerous and compact in layers 2 and 3. b and c: Higher magnifications of the region within the rectangle. **b**: Antibody Aβ_x-40_ stains the plaques, including their centres, relatively homogeneously. **c**: In a consecutive section abAβ_x-42_ leaves the centres pale disclosing the targetoid pattern of many plaques (arrows). In general the plaques are sharply delineated like the so-called cotton wool plaques (cf. Figure 7b and Additional file 1: Figure S9). Note the small diffuse Aβ deposits (arrowhead) and thin subpial patches (open arrow) in layer 1. **d**: In occipital cortex (striate area) the variable deposition of Aβ in different cortical layers is prominent. Note the abundant vascular Aβ deposition in meningeal and penetrating parenchymal arteries as well as in capillaries deep in layer 4. (See also Additional file 2: Figure S7c). **e**-**h**: Sections from the same region as in **a-c**. **e**: Mid-domain abAβ_17–24_ gives the most comprehensive staining of plaques. **f**-**h**: The more N-terminal antibodies highlight plaque centres. (*bar* in **a** 500 μm for **a** and **d**; *bar* in **b** 200 μm for **b**-**c** and **e**-**h**).

The area fraction of Aβ immunopositivity (Aβ_x-42_) in frontal, temporal and parietal cortices was about 25%. Plaques were present in all cortical layers, being most numerous and compact in layers 2 and 3, while in deeper layers they were somewhat larger and less distinct. In addition, in layer 1 there were often small diffuse plaques of variable numbers, shapes and staining intensities, as well as thin patchy variably immunopositive subpial bands (Figure 2a-h). The variation in the cortical pattern appeared e.g. in occipital cortex, where layers 4 and 6 were virtually devoid of plaques and layer 5 displayed only a lesser number of diffuse plaques (Figure 2d and Additional file 2: Figure S7c). Variation between patients was also observed e.g. as a paucity of plaques in layer 4 in frontal cortex of patients Am1 and Am2 compared to more abundant plaques in the same region in patients Sw1 and Sw2 (Additional file 3: Figure S1a-g, Additional file 4: Figure S2a, and Figure 2a).

#### Staining with general Aβ antibodies

With the C-terminal abAβ_x-42_ a majority of neocortical plaques in all four patients were ring-shaped (Figure 2c), as described in the first study on patient Sw1 [17], i.e. the weakly stained centres of larger plaques were surrounded by distinct immunoreactive coronas.

*In patient Sw1* the ring pattern visualized with abAβ_x-42_[11] was also clearly noticeable with abAβ_x-40_, abAβ_8–17_, abAβ_5–10_, and abAβ_arc_. The staining was progressively weaker and less distinct with the more N-terminal abs (Additional file 4: Figure S2a-g) and nearly negative with abAβ_1–5_, although with this antibody the small subpial plaques were still positive (Additional file 4: Figure S2f). No central accentuation with the N-terminal antibodies was observed (Additional file 4: Figure S2e-f).

**Figure 3:**
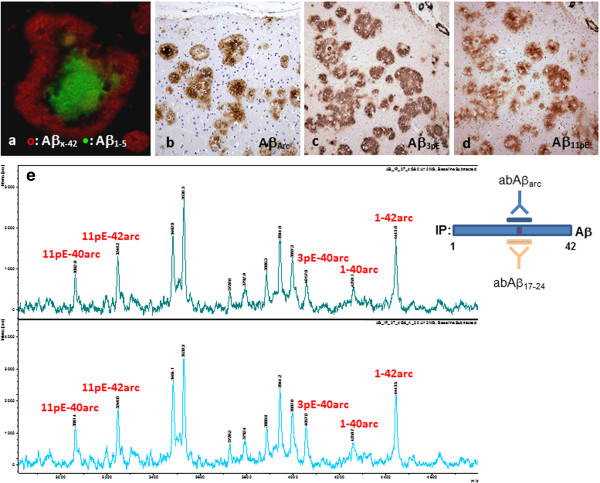
**Aβ immunostainings of Sw2 patient’s frontal and temporal cortex and mass spectra of Aβ peptides in Sw2 patient’s temporal cortex. a**: Confocal micrograph from Sw2 patient’s temporal cortex shows the targetoid pattern of the plaque: the corona contains Aβ_x-42_ and the centre Aβ_1-5_ species. **b**: The central accentuation of abAβ_arc_ (specific for the Arctic mutation) immunopositivity suggests that much of the mutated Aβ has preserved N-termini (Sw2, frontal cortex; see Figure 2g and h). **c** and **d**: Pyroglutamate specific abAβ_3pE_ and abAβ_11pE_ antibodies demonstrate that much of the Aβ is truncated and glutamates 3 and 11 are cyclised into pyroglutamate and this modification occurs in parallel at both sites. Note the strong positivity in pial arteries (Sw2, temporal cortex, consecutive sections). **e**: Average mass spectra of Aβ peptides extracted from Sw2 patient’s temporal cortex with formic acid and immunoprecipitated with abAβ_17–24_ and abAβ_Arc_ (conformation specific epitope 17-24arc), which precipitate both wild type Aβ and Arctic Aβ (see inserted cartoon). Three peaks corresponding to arcAβ species (with the Arctic mutation) and recognized by Aβ_pE_ specific antibodies (values observed in two independent MALDI-TOF experiments *m/z*=3061.5 and *m/z*=3062.8 Da → Aβ11pE-40arc; *m/z*=3246.2 and *m/z*=3246.0 Da → Aβ11pE-42arc; *m/z*=4057.0 Da → Aβ3pE-40arc) as well as two peaks corresponding to arcAβ with full length N-termini (*m/z*=4258.7 and m/z= 4259.1 Da → 1-40arc; *m/z*=4443.5 and m/z=4443.0 Da → 1-42arc) are labelled. Additional values with relative intensities of the peaks, see Additional file 5: Table S1. (*bar* in **a** 50 μm; *bar* in **b** 200 μm for **b**-**d**).

*In patients Sw2 and Am 1*, on the contrary, the neocortical plaques displayed a targetoid pattern: In these two brains the plaques were ring-shaped only with the most C-terminal antibody abAβ_x-42_ (Figure 2c)_,_ whereas with abAβ_x-40_ the centre was clearly immunopositive (Figure 2b). The mid-domain abAβ_17-24_ rendered the plaques most compact of all antibodies (Figure 2e), whereas antibodies with epitopes towards the N-terminus stained the plaque coronas more weakly and centres more intensely (Figure 2f-h, Additional file 3: Figure S1d-f). Confocal analysis of sections from patient Sw2 double immunostained with abAβ_x-42_ and abAβ_1-5_ clearly distinguished the two Aβ components in plaques: the peripheral corona was positive with abAβ_x-42_, while the centre was strongly positive with abAβ_1-5_ (Figure 3a). Therefore, the plaques could best be described as targetoid, not ring-shaped.

*In patient Am2* the staining pattern was more variable. In this patient’s frontal cortex it was similar as in patient Sw1, i.e. the more N-terminal abs also rendered the plaques ring shaped without intensely stained centres, although the staining was less distinct and much weaker (cf. Additional file 4: Figure S2). On the other hand, in Am2 patient’s temporal and occipital cortex many plaques displayed intensely stained centres, similarly to those found in patients Sw2 and Am1 (cf. Figure 2 and Additional file 3: Figure S1).

#### Staining with specific Aβ antibodies

*In patient Sw1* almost all plaques were ring-shaped with the Arctic specific antibody abAβ_arc_ (Additional file 4: Figure S2g). In patients Sw2, Am1 and Am2 the staining pattern with abAβ_arc_ resembled that with the mid-domain abAβ_17-24_, although it was somewhat weaker (Figures 3b vs. 2e and Additional file 3: Figure S1g vs. S1c).

*In patients Sw2 and Am1* the antibodies abAβ_3pE_ and abAβ_11pE_ showed that N-terminally truncated Aβ starting with pyroglutamate (Aβ_3pE_ and Aβ_11pE_) colocalized in frontal cortical plaques, although the staining with abAβ_11pE_ was weaker than with abAβ_3pE_ (for Sw2 Figure 3c-d and for Am 1 Additional file 3: Figure S1h-i). Aβ_3pE_ stained most plaques relatively homogeneously and much more extensively than the other N-terminal abAβ_1–5_ (cf. Figure 2h). Some strongly abAβ_1–5_ positive centres were also abAβ_3pE_ positive (Additional file 3: Figure S1h). Moreover, we observed vague predominant deposition of Aβ_11pE_ to the plaque centres (Figure 3d).

#### Correspondence between Aβ immunohistochemistry and mass spectrometry

Analysis by MALDI-TOF of Aβ peptides immunoprecipitated from Sw2 patient’s temporal cortex resulted in various peaks within the m/z range of 2000–5000 Da (Figure 3e and Additional file 5: Table S1). Noteworthy, several of the Aβ species (see labels) corresponded well with the predicted masses of Aβ_pE_ species, as detected by specific Aβ antibodies (abAβ_3pE_ and abAβ_11pE;_ Figure 3c-d). The observed and predicted m/z values with their relative intensities, corresponding to the peaks shown in Figure 3e are presented in Additional file 5: Table S1.

### Allocortical brain regions (phase 2)

#### Histopathology

Plaques in hippocampus were not as easily discernible with H&E staining as in neocortex–except for those located in dentate gyrus, where they were found to displace granular cells (Additional file 6: Figure S3a). In adjoining occipito-temporal cortex the pattern was similar as elsewhere in cerebral cortex. Although with Bielschowsky silver impregnation, both DNs and NFTs were strongly positive (Additional file 6: Figure S3b and inset), hippocampal plaques were virtually silver negative. However, in the same sections plaques in the nearby occipito-temporal cortex were clearly silver positive and the Aβ immunostainings of hippocampal plaques were intensely positive (see below). Congo red staining was negative (not shown).

#### Aβ immunohistochemistry

**Figure 4:**
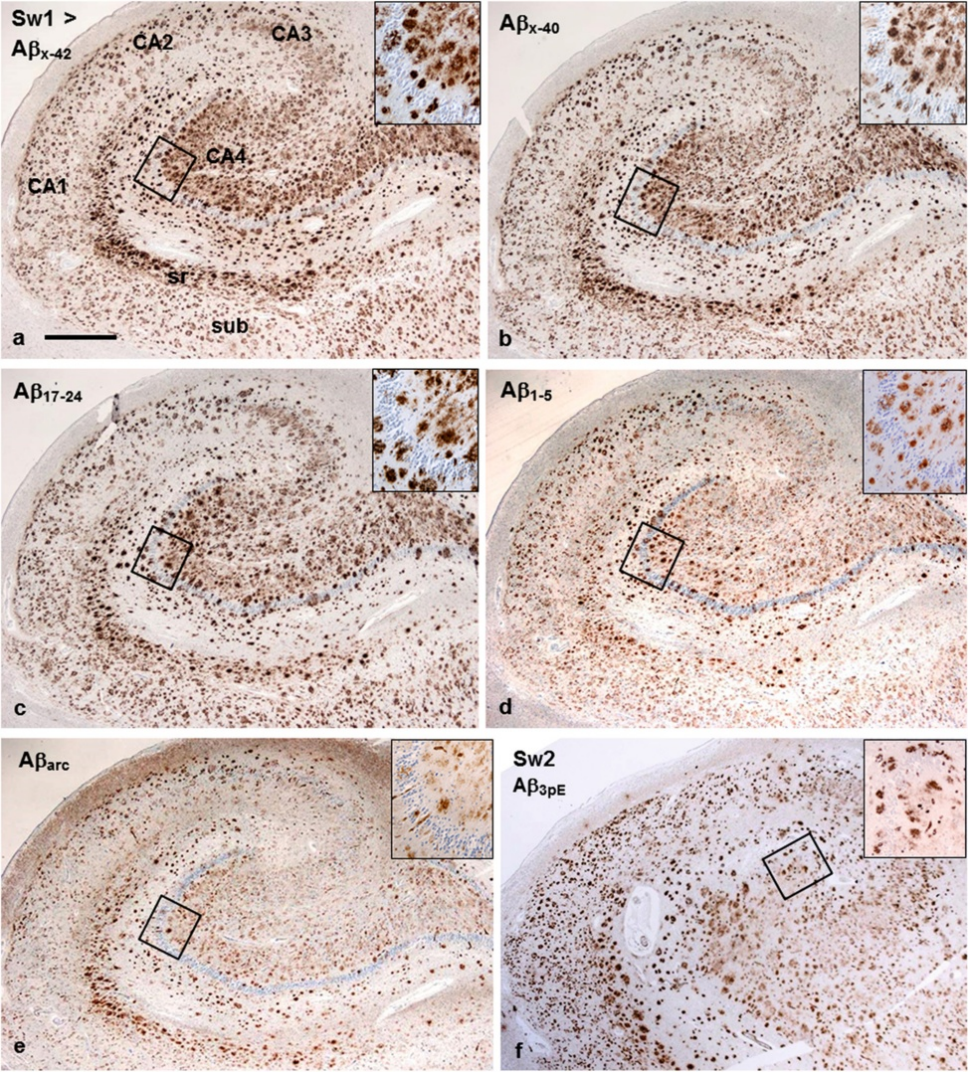
**Hippocampal region from patients Sw1 (a-e; consecutive sections) and Sw2 (f).** The general pattern of Aβ deposits is fairly similar with all antibodies, although the intensity of staining is somewhat stronger and the size of plaques larger with the C-terminal abAβ_x-42_**(a)** and abAβ_x-40_**(b)**, as well as with the mid-domain abAβ_17-24_**(c)** than with the N-terminal abAβ_1-5_**(d)** and the specific antibodies abAβ_arc_**(e)** and abAβ_3pE_**(f)**.The insets from the CA4 regions within the squares demonstrate that the plaques are mostly of diffuse type. In addition, especially in sectors CA3 and CA4, there is abundant, diffuse, lightly stained “background” deposition of Aβ. In stratum radiatum (sr) the plaques are somewhat larger and more compact. Note: no targetoid plaques with abAβ_x-42_ and abAβ_1-5_ (such as in cerebral cortex). (*bar* in **a** 1100 μm for all panels).

With Aβ immunostaining, the plaques were numerous throughout hippocampus, but their frequency and pattern varied in different hippocampal regions (Figure 4a-f).

In the CA1-CA4 sectors, the general Aβ antibodies disclosed abundant Aβ deposits of variable size and irregular shape. Remarkably, in hippocampus the plaques did not show such a distinct targetoid pattern as in cerebral cortex. Instead, they were small with a diffuse pattern when stained with different Aβ antibodies (insets of Figure 4a-f). In CA3 and CA4 sectors, in addition to the better defined plaques there were also background-like diffuse Aβ deposits, whereas in CA1 and CA2 such deposits were uncommon (Figure 4a-f).

Using the C-terminal abAβ_x-42_ (Figure 4a) and abAβ_x-40_ (Figure 4b), as well as the mid-domain abAβ_17–24_ (Figure 4c), the staining was recognizably stronger and the plaques were slightly more frequent than with the N-terminal antibodies abAβ_8–17_, abAβ_5–10_ and abAβ_1–5_ (Figure 4b). Diffuse plaques were also abundant in stratum radiatum, subiculum (Figure 4a-f) and transentorhinal cortex, whereas in entorhinal cortex they were relatively sparse. In the adjoining occipito-temporal cortex the plaques appeared similar as elsewhere in neocortex (cf. Figure 2a-h and Additional file 3: Figure S1 and Additional file 4: Figure S2).

Staining of allocortical sections with the specific Aβ antibodies abAβ_arc_ (Figure 4e), abAβ_3pE_ (Figure 4f) and abAβ_11pE_ (not shown) gave similar patterns as the general Aβ antibodies. The staining intensities were comparable to that of N-terminal abAβ_1−5_ (Figure 4d).

### Subcortical grey matter nuclei (phase 3)

#### Histopathology

In basal nuclei, plaques were most often not discernible with H&E or silver staining, but they were selectively positive for Aβ with immunohistochemistry (see below). In this region, the Congo red staining was negative in the parenchyma.

#### Aβ immunohistochemistry

Among the basal nuclei, claustrum was remarkably deviant: Aβ was deposited as large compact plaques, which had similar targetoid staining pattern as plaques in neocortex (Additional file 7: Figure S4a-g). On the contrary, in the neighbouring putamen the plaques were small and diffusely stained (Additional file 7: Figure S4h-k). These plaques were positive with the C-terminal abAβ_x-42_ (Additional file 7: Figure S4h) and abAβ_x-40,_ as well as with mid-domain abAβ_17-24_ (not shown) and were weakly positive with abAβ_arc_ (Additional file 7: Figure S4j) and abAβ_11pE_ (not shown). With the N-terminal abAβ_1-5_ plaques were almost negative (Additional file 7: Figure S4i), whereas with the pyroglutamate specific N-terminal abAβ_3pE_ the plaques were clearly discernible (Additional file 7: Figure S4k). In amygdala the Aβ deposition was similar to that in putamen, though with C-terminal antibodies the number of small diffuse plaques was greater (Additional file 7: Figure S4l). In thalamus (Additional file 7: Figure S4m) and caudate nucleus (not shown) the plaques were ragged and weakly stained with all antibodies. Globus pallidus was completely negative for Aβ-immunoreactivity (not shown).

### Brain stem (midbrain, pons and medulla; phase 4)

#### Histopathology

In midbrain, pons and medulla, Aβ deposits were not discernible with H&E and only weakly positive with silver staining. None of the parenchymal Aβ deposits were positive for Congo red (not shown).

#### Aβ immunohistochemistry

In midbrain, the deposition of Aβ was scarce. Diffuse weakly stained Aβ deposits were discernible almost exclusively in nucleus ruber. Among the different Aβ antibodies, only abAβ_x-42_ and abAβ_17–24_ stained these plaques. However, amyloid angiopathy could be clearly visualized with all Aβ antibodies (not shown). In pons, virtually no parenchymal deposits were observed despite brisk staining of blood vessels (not shown). In medulla, a few distinct plaques, strongly positive with all Aβ antibodies used were present in inferior olivary (Additional file 8: Figure S5a-i) and dorsal vagal nuclei (not shown). The pattern, number and size of plaques in medulla were approximately similar with both general and specific Aβ antibodies, although some variation in the intensity was observed (Additional file 8: Figure S5d-i). Remarkably, abAβ_x-42_ rendered the neuropil in inferior olivary nucleus distinctly positive (Additional file 8: Figure S5a and d), whereas with all other Aβ antibodies it was negative (e.g. Additional file 8: Figure S5b-c and e-i). Olivary neurons within the plaques appeared fairly well preserved (Additional file 8: Figure S5d-k), but both abAβ_x-42_ and abAβ_17–24_ stained cytoplasmic inclusions within inferior olivary neurons (Additional file 8: Figure S5d-e), as did also PAS and an antibody to lysosomal cathepsin D (Additional file 8: Figure S5j-k; see also paragraph *Intracellular Aβ immunoreactivity*).

### Cerebellum (phase 5)

#### Histopathology

In H&E stained sections the Aβ deposits were not detectable (not shown). Bielschowsky silver showed no impregnation in the Purkinje cell layer (see below) and only a small number of weakly positive perivascular streaks or smaller deposits in the molecular layer perpendicular to the surface (not shown). As elsewhere, Congo red did not reveal any parenchymal staining.

#### Aβ immunohistochemistry

The amount of Aβ deposited in cerebellum was much more abundant than that normally seen in AD. Furthermore, the pattern of the deposits was remarkably different from elsewhere in the Arctic AD patients’ brains, especially compared to the cerebral cortices. The immunopositive Aβ deposits were highly variable in size and had very irregular configurations, while distinct rounded Aβ plaques were completely absent. Furthermore, there were marked inter-individual differences, e.g. Aβ deposits in patient Am1 were distinctly different from those in patients Sw1, Sw2 and Am2 (see below).

**Figure 5:**
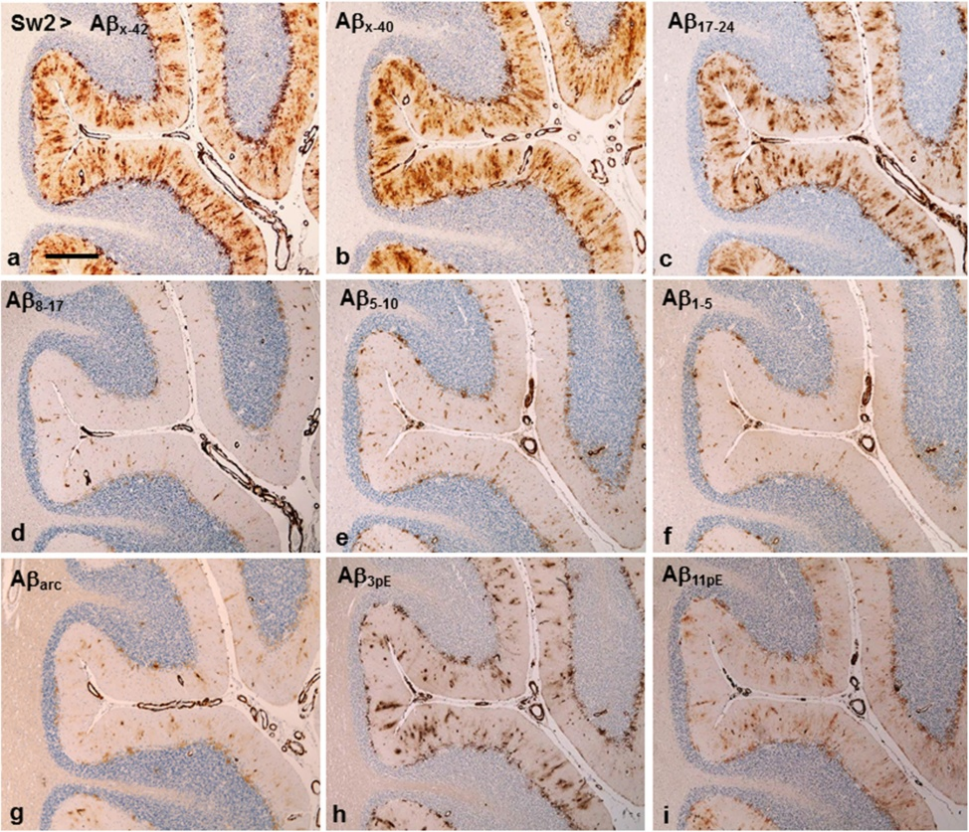
**Immunostainings of Sw2 patient’s cerebellum (a, c, d resp. e, f, h, i represent semiconsecutive sections). a**-**c**: C-terminal and mid-domain antibodies abAβ_x-42_, abAβ_x-40_ and abAβ_17–24_ disclose similar pattern. There is abundant deposition in the Purkinje cell layer, from where Aβ deposits extend as streaks towards the surface often loosely following penetrating arteries with distinct CAA. **d**-**i**: The more N-terminal (beyond aa 17) abAβ_8–17_**(d)**; abAβ_5–10_**(e)**; and abAβ_1–5_**(f)**, as well as the specific abAβ_arc_**(g)** render markedly weaker staining of the parenchymal deposits, which is also weaker than with the pyroglutamate specific abAβ_3pE_**(h)** and abAβ_11pE_**(i)**. With all Aβ antibodies blood vessels stain approximately as strongly. (*bar* in **a** 500 μm for all panels).

**Figure 6:**
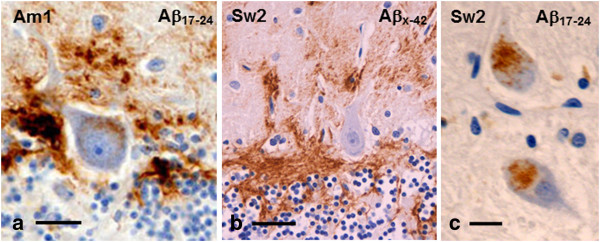
**Higher magnifications of the Purkinje cell layer from patients Am1 (a) and Sw2 (b) show the abundant Aβ deposition.** Granular cytoplasmic staining is observed with abAβ_17–24_ (most likely AβPP) in a Purkinje cell **(a)**, whereas with abAβ_x-42_**(b)** the cytoplasm is negative. Cytoplasmic abAβ_17–24_ positivity is also seen in Sw2 patient’s cerebellar dentate neurons **(c)**. (*bar* in **a** 30 μm; *bar* in **b** 50 μm; *bar* in **c** 15 μm).

*In patients Sw1, Sw2 and Am2* with similar Aβ staining pattern, the C-terminal abAβ_x-42_ (Figures 5a and 6b) and abAβ_x-40_ (Figure 5b) as well as the mid-domain abAβ_17–24_ (Figure 5c) displayed diffuse patchy staining in the Purkinje cell layer, from where irregular immunoreactive streaks extended across the molecular layer towards the surface, often following the penetrating blood vessels as wide and irregular cuffs (Figure 5a-c). A similar pattern, although with markedly weaker intensity, was obtained with the pyroglutamate specific abAβ_3pE_ (Figure 5h) and abAβ_11pE_ (Figure 5i). The staining was still weaker with the other N-terminal antibodies abAβ_8–17_, abAβ_5–10_ and abAβ_1–5_ (Figure 5d-f), as well as with abAβ_arc_ (Figure 5g). All Aβ antibodies used gave robust staining of blood vessel walls (Figure 5a-i).

*In Am1 patient* the staining pattern with abAβ_x-42_ and abAβ_17–24_ (Additional file 9: Figure S6a and c) was almost similar as in the three brains described above, whereas with the C-terminal abAβ_x-40_ (Additional file 9: Figure S6b) and mid-domain abAβ_8-17_ (Additional file 9: Figure S6d) both the deposits in the granular/Purkinje cell border zone and the streaks in the molecular layer were scarce and weak, although blood vessels were clearly positive. In this brain the more N-terminal abAβ_8–17_, abAβ_5–10_ and abAβ_1–5_ (Additional file 9: Figure S6d-f) stained almost exclusively arterial vessel walls. Among the specific antibodies the staining with Aβ_arc_ was faint, whereas both abAβ_3pE_ and abAβ_11pE_ gave clear staining Additional file 9: Figure S6g-i).

#### Intracellular Aβ immunoreactivity

In all brains definite cytoplasmic immunoreactivity was observed in inferior olivary neurons with abAβ_x-42_ and abAβ_17–24_ (Additional file 8: Figure S5d-e). The cytoplasmic Aβ(or AβPP [21]) immuno-positivity persisted, even if the formic acid pretreatment was omitted, whereas the extracellular Aβ deposits in the medulla were immunonegative (not shown). These granular cytoplasmic inclusions in inferior olivary neurons were also positive with PAS (Additional file 8: Figure S5j), cathepsin D (Additional file 8: Figure S5k), and α1-antitrypsin (not shown).

Cytoplasmic immunoreactivity with abAβ_17–24_ was observed also in some other locations, most prominently in cerebellum, both in Purkinje cells (Figure 6a) and in neurons of the dentate nucleus (Figure 6c). Markedly less intense staining was occasionally seen in cerebral cortical and hippocampal pyramidal neurons (not shown).

### Microscopy of cellular pathology

#### General alterations

**Figure 7:**
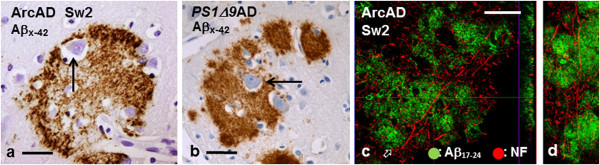
**Neurons and axons within neocortical Arctic Aβ plaques. a** and **b**: Sections of frontal cortex immunostained with the abAβ_x-42_ demonstrate occurrence of intact looking neuronal perikarya within a plaque (arrows) in Sw2 patient’s brain (**a**; see also Figure 8c) and within a cotton wool plaque in a *PS1Δ9* AD patient’s brain **(b)**. **c**: Representative confocal z-scan (monolayer; x–y; x–z and y–z projections) of a 50μm section from Sw2 patient’s temporal cortex displays numerous seemingly healthy neurofilament (NF) positive axonal structures (red) within and penetrating an abAβ_17–24_ positive Aβ deposit (green). **d**: A 50μm 3D projection of the region indicated by an open arrow in Figure 7c, also displays preserved axons within the Aβ deposits. (*bar* in **a** and **b** 40 μm; *bar* in **c** 40 μm for **c** and **d**).

In all Arctic AD patients’ brains neurons could be frequently identified within the cortical plaques. Interestingly, many of these neurons displayed relatively minor degenerative changes with characteristic vesicular nuclei and prominent nucleoli but had often somewhat condensed cytoplasm (Figure 7a). These neurons were similar as those found within the large cotton wool plaques in *PS1Δ9* AD patients (Figure 7b). Moreover, confocal microscopy analysis of cortical sections double-labelled for Aβ and neurofilament showed that some NF-positive axons traverse the plaques (Figure 7c-d). Similarly, Purkinje cells surrounded by abundant Aβ did not appear degenerating, not even those with intracellular abAβ_17–24_ positive deposits (Figure 6a-b).

#### Tau immunohistochemistry

**Figure 8:**
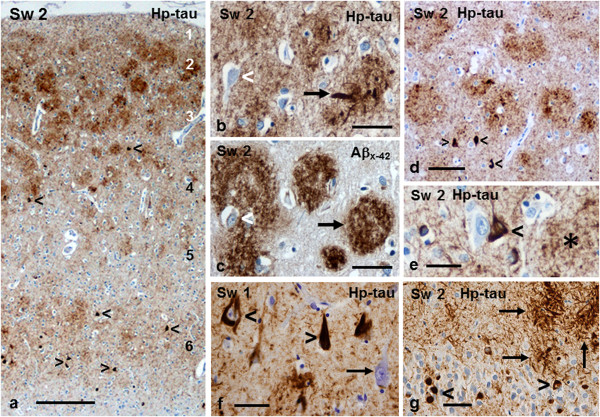
**Tau pathology in Sw2 patient’s frontal (a-c and e) and temporal (d) cortex. a**: Immunostaining for tau demonstrates abundant NTs, most prominent within Aβ plaques in layers 2 and 3, less numerous in layers 1, 4 and 6 and scarce in layer 5. Scattered small/atrophic neurons with NFTs are seen as dark spots (arrowheads). **b** and **c**: Consecutive sections demonstrate the accentuation of NTs within Aβx-42 positive plaques. A single plaque contains coarse DNs (arrow). The neuron within the large plaque (arrowhead) does not harbour a NFT. **d**: Neurons with NFTs (arrowheads) are predominantly located outside plaques. **e**: A neuron with an NFT (black arrowhead) is located outside the neighbouring plaque (asterisk) next to an hp-tau-negative neuron. **f**: Pyramidal neurons in CA1 of Sw1 patient’s hippocampus harbour prominent NFTs (arrowheads). Abundant NTs and a seemingly intact neuron (arrow) are noticeable in the surrounding tissue. **g**: Small granule cells in Sw2 patient’s dentate gyrus contain hp-tau-positive inclusions (arrowheads). Copious NTs and some coarser DNs (arrows) are seen in the adjoining hippocampal hilus. (*bar* in **a** 500 μm; *bar* in **b** 100 μm for **b-d**; *bar* in **e** 50 μm for **e-g**).

Hp-tau immunopositivity was abundant in cerebral cortices and it was mainly present as NTs. The frequency of NTs was accentuated within the Aβ plaques and thus plaques were delineated also by hp-tau staining (Figure 8a-g, Additional file 2: Figure S7a and c). Similar accentuation was also observed in *PS1Δ9* AD patients’ cotton wool plaques (Additional file 1: Figure S9i and j). NTs were usually abundant in cortical layers 2 and 3, markedly lesser in layer 4, scarce to absent in layer 5, and slightly accentuated in layer 6. Thus, the frequency of NTs by and large corresponded to that of compact Aβ plaques (Figure 8a-c; Additional file 2: Figure S7a and c). In calcarine cortex, the abundance of NTs corresponded to Braak stage VI, according to the BrainNet Europe recommendation (Additional file 2: Figure S7a). Within Aβ plaques NTs were usually delicate, but occasionally they appeared thicker approaching the appearance of DNs (Figure 8b and g).

Variable numbers of neurons with hp-tau positive NFTs were detected in all cerebral cortical areas examined (Figure 8a, d-e). Neurofibrillary deposits were found in the cytoplasm of small or atrophic neurons (Figure 8a, d-e), whereas larger (non-degenerated) neurons in cortical layers 3, 5 and 6, including those located within Aβ plaques, were usually devoid of NFTs (Figure 8b-c). Most commonly neurons with NFTs were not distributed within though nearby plaques (Figure 8d).

Many hippocampal pyramidal neurons from CA4 to subiculum as well as in adjoining entorhinal, transentorhinal and occipito-temporal cortices harboured prominent NFTs (Figure 8f). Furthermore, many of the granule cells in the dentate gyrus contained distinct hp-tau positive inclusions (Figure 8g). In general, NTs were abundant in the hippocampal neuropil and DNs were more prominent within hippocampal than cortical Aβ plaques (Figure 8g).

In claustrum, the pattern of NTs, DNs and neurons with NFTs was − like that of Aβ plaques − similar as in neocortex (cf. Figure 8a-b and d). In thalamus, where the Aβ plaques were small and diffuse, only few NFTs and very delicate NTs were discernible. In putamen, where plaques were even less conspicuous, almost no NTs were present (not shown). In globus pallidus, where no Aβ deposits were detected, neither were hp-tau immunoreactive structures seen (not shown).

Despite the abundance of Aβ deposits in cerebellar cortex and around Purkinje cells, no neurons (Purkinje cells, granule cells or other neurons) harboured NFTs. In addition, the number of NTs or DNs associated with Aβ deposits was insignificant (not shown).

#### Macro- and microglial changes

**Figure 9:**
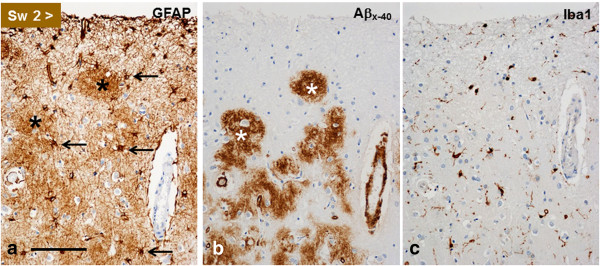
**Consecutive sections from Sw2 patient’s frontal cortex.** GFAP positivity **(a)** is accentuated within the Aβ_x-40_- positive plaques **(b)** and it appears to be due to denser network of processes, since astrocyte cell bodies (four marked with an arrow) are mainly located around or outside the plaques (asterisks). **c**: The Aβ deposition induces only slight and diffuse microglial (Iba1-positive) reaction, which does not seem to closely associate with the Aβ deposits in **(b)**. (*bar* in **a** 200 μm for all panels).

In all four brains, there was slight to moderate reactive astrogliosis in cerebral cortices. GFAP immunopositivity was accentuated within the Aβ-immmunoreactive plaques (Figure 9a-b), mainly as a meshwork of thin processes. Although the number of astrocytic cell bodies in cerebral cortices appeared to be slightly increased, they were relatively diffusely distributed instead of being strictly oriented within or around plaques (Figure 9a-b). Microglial reaction, as determined by Iba1 staining, in cerebral cortices was relatively modest and the distribution of microglial cells did not appear to follow the distribution of plaques (Figure 9b-c). However, their frequency seemed to vaguely correspond to the overall density of the NT meshwork (not shown).

In cerebellum there was pronounced GFAP staining around Purkinje cells (Bergmann astrocytes), although not as prominent as for Aβ. In the molecular layer GFAP positivity did not follow the perivascular streaks of Aβ deposits. Instead, loose GFAP-immunopositive astrocytic network of varying intensity was found throughout the molecular layer (Additional file 10: Figure S8a-b). In all patients, the Iba-1 immunoreactivity in cerebellum was weak and diffuse compared to the Aβ deposition and astroglial reaction and it did not specifically associate with Aβ deposits (Additional file 10: Figure S8c).

#### Vascular pathology

No major atherosclerosis in the circle of Willis was present, neither were there infarcts, haemorrhages nor microbleeds.

In all four patients’ brains leptomeningeal and cortical penetrating arteries of widely different calibres were variably immunopositive with the different Aβ antibodies used (e.g. Figures 2d and g, 5a-i and Additional file 9: Figure S6a-i). In addition, capillaries in many particular anatomic locations were positive for Aβ, most commonly with abAβ_x-40_ (Figure 2d and Additional file 2: Figure S7c). Presence of fibrillar Aβ within the vessel walls, i.e. evidence of true cerebral amyloid angiopathy (CAA), was verified by Congo positivity with green birefringence in arteries, but not in capillaries (Figure 1d, inset). Details of the composition, distribution and severity as well as illustrations of CAA in these Arctic AD patients’ brains will be presented in a separate article (in preparation).

## Discussion

### Fulfilling the neuropathological criteria of AD

We performed neuropathological examinations of the brains from four patients who carried the *Arctic AβPP* mutation and whose clinical picture complies with AD. We could demonstrate that the neuropathological hallmarks of AD, Aβ plaques and deposition of hp-tau, were prominent features in all four patients’ brains. However, the Arctic AD pathology has certain features that deviate from the common AD pathology [22], as it is referred to in both of the latest consensus criteria [23-25].

When the ABC system of the new 2012 NIA-AA criteria [23,25] is applied, the distribution of Aβ plaques, i.e. NIA-AA component A = 3, since the accumulation of Aβ is florid even in cerebellum (= Thal phase 5, [20]). As for the neurofibrillary tau pathology (component B), the presence of NFTs throughout the neocortical regions fulfils the criteria of Braak stage V-VI [26], i.e. NIA-AA component B = 3. Braak stage V-VI is also met, if the Brain Net Europe (BNE) staging is applied [27], as the neocortical NTs are abundant in the striate area (occipital cortex; Additional file 2: Figure S7a). The grading of component C in the Arctic brains according to CERAD as recommended in NIA-AA criteria is somewhat problematic, because of the exceptional plaque structure. Although Aβ plaques are compact, they are devoid of fibrillar amyloid cores and only rarely harbour robust DNs. Nevertheless, we propose that they do fulfil the CERAD criteria for neuritic plaques, since they display delicate hp-tau positive NTs and occasional DNs. The frequency of plaques should be considered frequent, i.e. the component C = 3 [28].

Taken together, our neuropathological findings in the Arctic AD patients’ brains meet both the 2012 NIA-AA criteria for high level of AD- related changes (A3, B3, C3) [23,25] and the previous NIA-RI criteria for a high likelihood of AD [24]. The very rare α-synuclein positive neurons encountered (not shown) most likely did not contribute to the patients’ clinical picture. Neither did we find any significant ischemic pathology, although CAA was relatively prominent. Thus, the dementia caused by the *Arctic AβPP* mutation is due to AD, albeit with unique features of AD neuropathology.

Interestingly, the large, round cortical Arctic plaques bear resemblance to the cotton wool plaques, such as the Aβ deposits found in AD patients with *PS1* mutations [2-4]. Similarly, the cotton wool plaques and the Arctic plaques are devoid of amyloid cores and harbour very few hp-tau-positive DNs [3]. However, cotton wool plaques have a homogeneous composition (Additional file 1: Figure S9), whereas the Arctic plaques are composed of variably truncated and spatially differentially distributed Aβ and therefore when immunostained with different Aβ antibodies they appear targetoid.

### Amount of Aβ deposition

The extent of Aβ deposits in the Arctic AD patients’ brains was massive, which may explain why the weights of Sw1, Sw2 and Am2 brains were considerably higher than what is commonly recorded in sporadic AD. Alzheimer brains are usually atrophic, but no studies have systematically compared the brain weight with the Aβ burden. Interestingly, the brain weights in three of the five *PS1Δ9* AD patients with large cotton wool plaques of similar abundance as Aβ plaques in our Arctic AD patients’ brains were within normal limits [3].

### Hierarchical order of Aβ deposition

In sporadic AD, Aβ deposition has been suggested to occur hierarchically in five phases [20]. The distribution of Aβ deposits in the Arctic AD patients’ brains corresponds to Thal’s most advanced phase 5, for which the requirement is the presence of Aβ also in cerebellum. Aβ deposits were highly abundant in all Arctic AD patients’ cerebellum, but they were few in brain stem nuclei–the criterion for phase 4. These features suggest that, in these brains, cerebellum may have become involved at an earlier phase or at a faster pace than the brain stem. Thus, the *Arctic AβPP* mutation may alter the hierarchy according to which the Aβ deposits are generally seen to emerge in AD patients’ brains. Perhaps abundant extracellular perivascular Aβ aggregates in Arctic AD brain overwhelm the perivascular drainage pathways [29] in cerebellum leading to an early pathology. Moreover, the pattern of Aβ deposition and distribution in our Arctic patients’ cerebellum was exceptional (see next section).

### Variation in the distribution of Aβ deposition

The pattern and composition of Aβ deposits in the Arctic AD brain revealed additional topographic variability. The ring plaques, originally discovered by Bielschowsky silver and immunostaining with abAβ_x-42_[17], were − with the use of additional Aβ antibodies − shown to be targetoid rather than ring-shaped (as preliminarily demonstrated in our previous article [18] and now in greater detail in this article). These plaques were almost only observed in cerebral cortex, whereas in other anatomical locations the plaques were usually of more irregular and diffuse types.

Interestingly, in claustrum the pattern of Aβ deposits was similar to that in cerebral cortex. This is consistent with a previous observation of cotton wool plaques in *PS1Δ9* AD patients’ brains [30]. In addition, the tau pathology in claustrum was similar as in cerebral cortex. Claustrum is generally considered to be a part of the basal ganglia and the presence of plaques in this region would thus correspond to Thal’s phase 3. However, this pattern was strikingly different from that in the other subcortical grey matter nuclei. For example, globus pallidus was virtually negative for Aβ plaques, while only small diffuse plaques could be found in putamen, thalamus and caudate nucleus. This discrepancy may be related to the fact that, of these regions, only claustrum has bidirectional connections with almost all cortical regions [31].

The pattern of cerebellar Aβ deposits differed remarkably from the commonly observed restriction of deposits to the molecular layer in sporadic AD [20] and from the abundance of cored plaques in *PS1Δ9* AD patients’ cerebellum. In the Arctic AD patients’ brains, the abundant Aβ deposition next to the Purkinje cells may indicate active Aβ production in these cells. Moreover, the apparent pattern of Aβ deposition along the perivascular drainage pathways (also depicted in NIA-AA article by Hyman et al. 2012 [23]) may be explained by a relatively profuse transport of extracellular Aβ through the molecular layer to the subarachnoid space [29].

### Differential truncation of Aβ

The composition of Aβ deposits in both sporadic and familial AD brains has been shown to be highly variable, with both N- and C-terminal truncations and modifications [32-37].

Our mass spectrometric analyses, in which we immunoprecipitated both wt and mutated Aβ from the temporal cortex of the patient Sw2 with antibodies against two distinct Aβ epitopes (ArcAβ_17–24_ and wtAβ_17–24_), demonstrated that the deposits contained variably truncated and modified Aβ species, both wt and Arctic Aβ. Furthermore, our immunohistochemical stainings revealed that although all four patients carried the same *AβPP* mutation, both the distribution and the composition (truncations or modifications) of the Aβ deposits showed considerable inter- and intra-individual variability. This should not actually be surprising, since it is most likely that the “machinery and milieu” in the AβPP production, processing and/or Aβ aggregation are not identical in the four individual Arctic AD patients, nor in different anatomic regions within each patient’s brain. Whether a similar variability in the composition of Aβ deposits exists in other forms of FAD has been the subject of several studies [5,34-37].

The variability in the composition of deposited Aβ also sets new requirements for the antibodies used in diagnostic work. Since truncations can occur at many different sites of the Aβ peptide, some antibodies give virtually negative parenchymal staining. Thus, either an antibody against the mid-portion of Aβ preferably against an epitope located C-terminally to aa 11 (to avoid problems with 11pE truncated Aβ species) and N-terminally to aa 40 (to avoid problems with the different C-terminal truncations), or a mixture of different antibodies should be recommended for routine analyses. In our hands, the antibody abAβ_17–24_ (clone 4G8) appeared to best fulfil such requirements (even though it also recognizes *AβPP*[21]), whereas the other widely used mid-portion antibody abAβ_8–17_ (clone 6F/3D) yielded inconsistent results. For instance, it gave only vague staining of the cerebellar parenchymal deposits, although it stained blood vessels in this region clearly positively.

It is known that the Arctic mutation interferes with the processing of AβPP by making it less prone for α-secretase cleavage, while elevating β-secretase cleaved fragments [12,38]. Natural processing of AβPP at the β′-site (at Aβ aa 10/11) is also favoured over the β-site (at Aβ aa −1/1) in situations when accession to it by BACE1 enzyme is affected, e.g. through structural twists in AβPP [39]. Moreover, N-terminally truncated and modified Aβ peptides (e.g. AβpE3-x and AβpE11-x) have been shown to be significantly increased in the brains of AD patients with various PS1 mutations [36,40]. Our data show that the Arctic mutation like the PS1 mutations referred to above may increase cleavages at aa 3 and 11 since we observed 3pE40arc, 11pE40arc and 11pE42arc in our MS measurements (Figure 3e, Additional file 5: Table S1 and [18]), and considerable immunopositivities with ab3pE and ab11pE antibodies. However, since we also observed a heterogeneous population of N-terminally truncated Aβ peptides, additional (primary/secondary) cleavages are also likely to occur in the Arctic AD brain. Aβ-peptides might also aggregate in a way that exposes cleavage sites and facilitates peptide truncation and their modifications as a secondary process.

Lack of fibrillar Aβ and amyloid cores is a characteristic feature of both Arctic plaques and *PS1Δ9* cotton wool plaques. The reason for this feature in these two genetic variants of AD is unknown. Certain properties of the mutated forms of Aβ such as posttranslational modifications and altered propensity to oligomerize/aggregate, could offer an explanation for the limited formation of structurally ordered Aβ fibrils in Arctic AD patients [41], whereas the underlying reason in the *PS1Δ9* AD patients is even less clear, because the cotton wool plaques in such brains contain wild-type Aβ only. It might be that in these two types of FAD Aβ aggregation is too rapid and does not lead to Congo-red positive, fibrillar Aβ deposits.

It has been shown that the Arctic mutation leads to an accelerated oligomerization and disordered fibrillogenesis of Aβ, measured both *in vitro*[11,41-44] and *in vivo*[11,45,46]. The diameter of Arctic Aβ fibrils correlated with decreased neuronal viability [42]. Recent *in vitro* experiments on the aggregation process of ArcAβ_1–40_[41] demonstrated that at least four types of fibrils can be identified. The intermediate phase of spherical aggregates appeared at earlier time points and ArcAβ_1–40_ fibrils polymerized more rapidly and at lower concentrations than wt Aβ_1–40_ fibrils. At late stages fragmentation and clustering of ArcAβ_1–40_, but not of wtAβ_1–40,_ fibrils were observed [41]. The results of these experiments are in agreement with the suggestion that spherical aggregates (containing abundant β-hairpin and/or β-sheet structures), and/or Aβ oligomers have a pathogenic role in the AD brain. Especially the larger soluble oligomers, i.e. protofibrils, are known to have neurotoxic properties. However, an alternative Aβ aggregation pathway, different from simple assembly of spherical aggregates and protofilaments into fibrils, has also been proposed [41], which may contribute to the distinct morphology of Aβ plaques in Arctic AD patients (i.e. Congo-red negativity of the Arctic AD deposits). Moreover, co-incubation of ArcAβ with wtAβ_1-40_ led to kinetic stabilization of Arctic protofibrils [47]. An increase in the ratio of ArcAβ to wtAβ in Arctic AD may result in the rapid accumulation of neurotoxic protofibrils and acceleration of the disease process [48].

### Cellular pathologies

The large plaques in both the Arctic and *PS1Δ9* AD patients’ brains were found to embrace neurons. More specifically, in all four Arctic brains seemingly viable neocortical pyramidal neurons and cerebellar Purkinje cells could be identified within several plaques of variable Aβ composition. Interestingly, the perikarya of these neurons seemed intact and did not appear to be under way to develop neurofibrillary pathology. This sparing of neuronal perikarya is in accordance with the notion that intraneuronal Aβ triggers neuron loss in AD [49]. In several animal models it has been demonstrated that extracellular Aβ plaques do not seem to instigate neuronal death, whereas accumulation of intraneuronal Aβ correlated well with the loss of neurons [50], including a transgenic model expressing pyroglutamate Aβ_3pE-42_[51].

We showed that even apparently intact axons traversed the plaques. These observations are phenomena, similar to those in *PS1Δ9* AD patients, i.e. axons are not pushed aside to wind around the accumulated Aβ. However, the relatively low number of neurofilament positive intraplaque axons may indicate that axons traversing Arc plaques suffer from some degree of degeneration, as it was interpreted to occur within *PS1Δ9* AD patients’ cotton wool plaques [3]. The intraplaque accentuation of NTs (i.e. axons containing hp-tau) in both Arc and *PS1Δ9* plaques supports the suspicion of axonal degeneration. Accumulation of pathogenic species of microtubule associated tau protein can impair axoplasmic transport and consequently contribute to synaptic loss, which may be pivotal in the pathogenesis of AD [52]. If the synaptic contacts are lost, sparing of the perikarya or axons *en route* cannot prevent functional loss (and further degeneration). The fate of axons within Aβ plaques obviously merits further analysis.

Furthermore, the non-fibrillar type of Aβ deposits in the Arctic brain induced only a limited reactive response. Although the density of astrocytic processes was increased within the Arctic Aβ deposits (clearly visible around Purkinje cells), the astrocytic cell bodies appeared not to cluster around the non-fibrillar Aβ deposits. Likewise, in *PS1Δ9* AD patients’ brains astrocytes did not cluster around the non-fibrillar cotton wool plaques, although in these brains the scarce cored plaques were surrounded by an increased number of astrocytes [3]. Similarly, the Arctic non-fibrillar plaques did not appear to attract microglial cells. The presence of preserved neuronal perikarya and axons as well as the lack of activated glial cells strengthen the perception of extracellular non-fibrillar Aβ deposits as being relatively non-toxic. Thus, it is conceivable that Aβ oligomers, which are considered pathogenic, exert their effects already within the neurons or by being diffusely distributed (not in plaques) in the parenchyma.

## Conclusions

We have demonstrated special neuropathologic characteristics in the brains of four deceased AD patients with the *Arctic AβPP* mutation (p.E693G/p.E22G). The amount of Aβ deposited in the brains was profuse, but virtually all parenchymal deposits were composed of non-fibrillar, Congo negative Aβ aggregates Aβ Congo red only stained the walls of moderately to severely angiopathic vessels. Mass spectrometric analyses on temporal cortex samples showed that the Aβ deposits contained variably truncated and modified wt and mutated ArcAβ species. The structure and composition of the Aβ deposits varied considerably between the patients. In three of the four analysed Arctic AD brains, the plaque centres containing C-terminally (beyond aa 40) and variably N-terminally truncated Aβ were surrounded by Aβ_x-42_ immunopositive coronas giving the plaques a targetoid appearance. Furthermore, in each individual the architectural pattern of plaques was found to vary between different anatomic regions–from diffuse deposits to targetoid or ring-shaped plaques. Tau pathology appeared mainly as delicate neuropil threads with accentuation within Aβ plaques. Thicker dystrophic neurites were only occasionally observed. Neurons within the large Aβ plaques appeared relatively intact. Thus, the extracellular deposits of non-fibrillar Aβ did not seem to directly damage neuronal perikarya or to induce formation of neurofibrillary tangles, supporting the present view of intracellular Aβ oligomers being neurotoxic. The enrichment of NTs within plaques may indicate axonal damage, even though neurofilament positive axons traversing plaques were detected. Finally, similarly as the cotton wool plaques in *PS1*Δ9 AD, the Arctic plaques induced only a modest glial and inflammatory tissue reaction.

## Availability of supporting data

The supplemental figures (named as Additional files 1, 2, 3, 4 and 6, 7, 8, 9, 10) and supplemental table (named as Additional file 5) supporting the results of this article are included within the article.

## Abbreviations

Aβ: Amyloid-β; AβPP: Amyloid-β precursor protein; abAβ: Antibody to indicated amyloid-β; AD: Alzheimer’s disease; BNE: Brain Net Europe; CERAD: The Consortium to Establish a Registry for Alzheimer’s disease; CAA: Cerebral amyloid angiopathy; DN: Dystrophic neurite; ELISA: Enzyme linked immunosorbent assay; FAD: Familial Alzheimer’s disease; GFAP: Glial fibrillary acidic protein; hp-tau: Hyperphosphorylated tau; MALDI-TOF: Matrix-assisted laser desorption/ionization-time of flight mass spectrometry; NFT: Neurofibrillary tangle; NIA-AA: National Institute on Aging and Alzheimer’s Association; NT: Neuropil threads; PAS: Periodic acid schiff; PIB-PET: Pittsburgh compound B-positron emission tomography; PS1Δ9: Presenilin 1 with deletion of exon 9-mutation; Wt: Wild type.

## Competing interests

The authors declare that they have no competing interests.

## Authors’ contributions

HK: was responsible for the histopathology and immunohistochemistry analyses, designed the study and was a primary author of the manuscript. ML: analyzed the mass spectroscopic data and participated in the design and writing and composition of the pictorial material of the ms. NB: performed the autopsy and primary histopathological and immunohistochemical evaluation of the patient Sw1. OP: performed biochemical and confocal microscopic analyses of the patient Sw2. TDB: Performed the clinical evaluation of patients Am 1. DN: Performed the neuropathological evaluation of patient Am 1. GDS: Performed the neuropathological and genetic analyses of Am1 and Am2. RMB: Performed the clinical evaluation of patient Sw2. TO: performed the autopsy and primary histopathological and immunohistochemical analyses of the patient Sw2. RS: has performed the MALDI-MS measurements and analyzed the mass spectrometry data. MB participated in the design and writing and composition of the pictorial material of the ms. OW: performed the pyroglutamate immunohistochemistry and participated in the composition of the ms. TAB: performed the pyroglutamate immunohistochemistry and participated in the composition of the ms. LGN: supervised and performed biochemical analyses of the patient Sw2 and participated in the design, coordination and composition of the manuscript. HB: Performed the clinical evaluation of patients Sw 1 and Sw2. LL: Performed the clinical evaluation of patients Sw 1 and Sw2. MI: Performed the clinical analyses of patient Sw2, coordinated the design of the study and participated in the composition of the ms. All authors have read and approved the contents of the manuscript.

## Authors’ information

Hannu Kalimo and Maciej Lalowski: These authors share the first authorship.

## Supplementary Material

Additional file 1: Figure S9 Immunostainings of semiconsecutive sections from a PS1Δ9 AD patient’s frontal (ah) and temporal (i and j) cortex. The cotton wool plaques are similarly rounded structures as Arctic plaques, but the different antibodies stain them homogeneously, although with different intensities. The strong staining with abAβ_x-42_ (a) suggests that majority of Aβ terminates at aa 42, whereas Aβ_x-40_ (b) species are scarce. N-termini appear to be markedly variable including considerable amounts of N-terminally truncated Aβ species starting with pyroglutamate (Aβ_11pE_ or Aβ_3pE_; g and h). The homogeneous staining suggests that the variably truncated Aβ species are relatively evenly distributed within the plaques. i and j: Sections from temporal cortex show accumulation of hp-tau positive NTs within Aβ plaques (six plaques marked with numbers). Note the NTs (arrows) also in the subpial Aβ positive edging (asterisks) (bar in a 100 μm for a-h; bar in i 150 μm for i and j). Click here for file

Additional file 2: Figure S7 a: Sw2 patient’s striate area (visual cortex). The marked density of hp-tau positive NTs, including moderate density in layer 5, qualifies for the Braak stage VI of BrainNet Europe recommendation. The distribution of NTs by and large corresponds to that of Aβ plaques in the consecutive section c (asterisks). Note also the numerous Aβ-positive capillaries in layers 4–5. LFB-CV = Luxol fast blue-cresyl violet. (See also Figure 2d) (*bar* in a 400 μm for all panels). Click here for file

Additional file 3: Figure S1 In semiconsecutive sections from Am1 patient’s frontal cortex the plaques appear ring-shaped with C-terminal abAβ_x-42_ and abAβ_x-40_, mid-domain abAβ_17-24_ and Arctic specific abAβ_arc_ (a-c and g plus insets), whereas with more N-terminal (beyond aa 17) abAβ_8-17_, abAβ_5-10_ and abAβ_1-5_ (d-f) the plaques stain progressively weaker, although some plaques have intensely stained centres. Robust staining with abAβ_arc_ suggests an abundance of Arctic Aβ and a fair amount of Aβ appears to have pyroglutamate at positions 3 and 11 (h and i). The lesser number of plaques in layer 4 creates a sparsely populated band, best visible in b-d and g-i (similar band was observed in Am2 patient). No plaques are detectable in layer 1. Leptomeningeal blood vessels in b-i are strongly Aβ-positive, whereas in a they are only weakly positive. (*bar* in a 300 μm for all panels). Click here for file

Additional file 4: Figure S2 Semiconsecutive sections from Sw1 patient’s frontal cortex. All antibodies except for abAβ_1-5_ disclose ring-shaped plaques (insets and arrows) in layers 2–6. The small subpial plaques in layer 1 are of diffuse type. Contrary to findings in patients Sw2 (Figure 4) and Am1 (Additional file 3: Figure S1) plaque centres are not intensely stained (*bar* in a 300 μm for all panels). Click here for file

Additional file 5: Table S1 Aβ peptides detected in MALDI-TOF analyzes of immunoprecipitates from temporal cortex of Arctic AD Sw2 patient brain. Legend: Aβ peptides were measured in positive linear mode on AutoflexIII imager Bruker MALDI-TOF spectrometer in two independent experiments (named as exp1 and exp2). All *m/z* listed represent [Mav+H]+ (± 3 Da), after smoothing and background subtraction. Extraction of Aβ peptides was performed in 99% formic acid. Immunoprecipitation (IP) was performed with abAβ17-24 (Mab4G8) and abAβarc (Mab27) antibodies. Asterisks mark peaks labeled in Figure 3e. Molecular weight (m/z) in Daltons (Da). Relative intensities of the peaks in relation to the most intensive peak (Aβ17-43wt) were calculated with FlexAnalysis 3.4 software (Bruker Daltonics). The data from experiment 1 were presented in Philipson et al. *Neurobiol Aging* (2012), 33: 1010 e1011-1013 [18]. Click here for file

Additional file 6: Figure S3 Sw2 patient’s hippocampus. a: In H&E stained sections the plaques are difficult to discern, except when they are located within the dentate gyrus displacing granule cells (arrows). b: With Bielschowsky silver impregnation DNs within plaques are clearly visible, whereas plaques themselves are inconspicuous. Inset: NFT in a hippocampal pyramidal neuron is strongly silver positive. (*bar* in a 100 μm for a and b). Click here for file

Additional file 7: Figure S4 a-g: Aβ-plaques in Swe2 patient’s claustrum show similar targetoid pattern as in neocortex (a-c consecutive sections). a: With abAβ_x-42_ dark corona and pale centre. b: With abAβ_x-40_ fair staining of both centre and corona. c: With abAβ_1-5_ dark centre and pale corona. d: Middomain abAβ_17-24_ stains strongly both centre and corona. e: Specific abAβ_arc_. gives similar pattern as abAβ_17-24_, though with much lesser intensity. f and g: Plaques comprise of both Aβ_3pE_ and Aβ_11pE_, though less of the latter. h-k: Plaques in Sw2 patient’s putamen are small and diffusely stained. The most intense stainings are seen with abAβ_x-42_, abAβ_arc_ and abAβ3_pE_ (h, j and k) suggesting an abundance of Aβ with pyroglutamate-modified N-termini, which is consistent with the virtually negative abAβ_1-5_ staining (i). l: In Sw2 patient’s amygdala plaques are similar as in putamen but more numerous. m: In Sw2 patient’s thalamus the plaques are ragged and weakly stained. (*bar* in a 100 μm for a-c; *bar* in d 100 μm for d-g; *bar* in h 50 μm for h-l; *bar* in m 50 μm). Click here for file

Additional file 8: Figure S5 a-k: Sw2 patient’s medulla. A few compact plaques are positive with C-terminal (a), mid-domain (b) and N-terminal antibodies (c). Remarkably, abAβ_x-42_ renders the neuropil in inferior olivary nucleus distinctly positive (a and d), whereas with the other antibodies it is negative (b, c and e-i). Both abAβ_x-42_ and abAβ_17-24_, (d and e; arrows), but not the rest of Aβ antibodies applied (f-i), stain granular inclusions in the cytoplasm of seemingly well preserved olivary neurons within and adjacent to plaques. The neuronal inclusions also stain with PAS (j) and an antibody to lysosomal cathepsin D (k). (*bar* in a 350 μm for a-c; *bar* in d 50 μm for d-i; *bar* in j 25 μm; *bar* in k 30 μm). Click here for file

Additional file 9: Figure S6 a-i: Immunostainings of Am1 patient’s cerebellum demonstrates the marked interindividual variation despite the same genetic defect (cf. Figure 5). Only abAβ_x-42_ and abAβ_17-24_ are clearly positive (a and c), whereas abAβ_x-40_ (b) and the more N-terminal abAβ_8-17_, abAβ_5-10_ and abAβ_1-5_ (d-f) give virtually no parenchymal staining, even though the blood vessels are strongly positive. Weak staining with abAβ_arc_ (g) is consistent with most parenchymal deposits being composed of wild-type Aβ. A fair proportion of the deposited Aβ appears to have pyroglutamate N-termini (h and i). (*bar* in a 150 μm for all panels). Click here for file

Additional file 10: Figure S8 Sw2 patient’s cerebellum. a: The density of GFAP-positive network is prominent, especially in the molecular layer. b: The density of the network corresponds to the deposition of Aβ in the Purkinje cell layer, but it differs from the perivascular accentuation of Aβ in the molecular layer. c: The microglial reaction to Aβ is minimal (*bar* in a 200 μm for all panels). Click here for file
