# Supplementary material for: The Arctic AβPP mutation leads to Alzheimer’s disease pathology with highly variable topographic deposition of differentially truncated Aβ
Source: Acta Neuropathol Commun. 2013 Sep 10;1:60. doi: 10.1186/2051-5960-1-60 (PMC4226306; doi:10.1186/2051-5960-1-60)
Supplement: Additional file 1: Figure S9 — Immunostainings of semiconsecutive sections from a PS1Δ9 AD patient’s frontal (ah) and temporal (i and j) cortex. The cotton wool plaques are similarly rounded structures as Arctic plaques, but the different antibodies stain them homogeneously, although with different intensities. The strong staining with abAβx-42 (a) suggests that majority of Aβ terminates at aa 42, whereas Aβx-40 (b) species are scarce. N-termini appear to be markedly variable including considerable amounts of N-terminally truncated Aβ species starting with pyroglutamate (Aβ11pE or Aβ3pE; g and h). The homogeneous staining suggests that the variably truncated Aβ species are relatively evenly distributed within the plaques. i and j: Sections from temporal cortex show accumulation of hp-tau positive NTs within Aβ plaques (six plaques marked with numbers). Note the NTs (arrows) also in the subpial Aβ positive edging (asterisks) (bar in a 100 μm for a-h; bar in i 150 μm for i and j). [file 2051-5960-1-60-S1.pdf]

Title: The *Arctic APP* mutation leads to Alzheimer's disease pathology with highly variable topographic deposition of differentially truncated Aβ

Journal: Acta Neuropathologica Communications

Authors: Hannu Kalimo[1], Maciej Lalowski, Nenad Bogdanovic, Ola Philipson, Thomas D. Bird , David Nochlin, Gerard D. Schellenberg, RoseMarie Brundin, Tommie Olofsson, Marc Baumann, Oliver Wirths, Thomas A. Bayer, Lars N.G. Nilsson, Hans Basun, Lars Lannfelt, Martin Ingelsson

Corresponding author: [1]Hannu Kalimo, Department of Pathology, University and University Hospital of Helsinki, Helsinki, Finland,

E-mail: hannu.kalimo@helsinki.fi

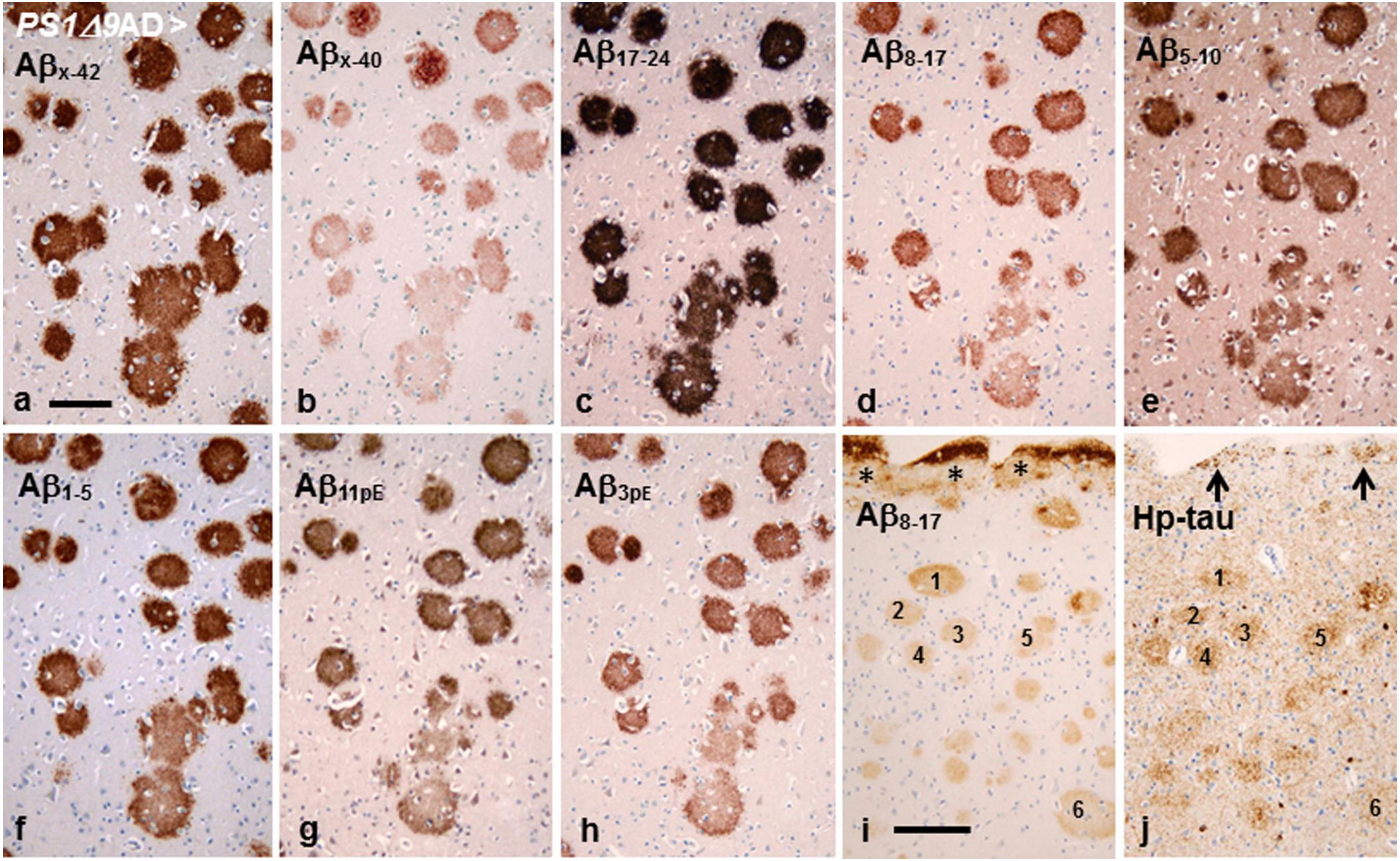

PS1Δ9AD >
Aβx-42
a
Aβx-40
b
Aβ17-24
c
Aβ8-17
d
Aβ5-10
e
Aβ1-5
f
Aβ11pE
g
Aβ3pE
h
*
*
*
Aβ8-17
1
2
3
4
5
6
i
Hp-tau
1
2
3
4
5
6
j

Suppl. Fig. 9 Immunostainings of semiconsecutive sections from a *PS1*Δ9 AD patient´s frontal (**a-h**) and temporal (**i** and **j**) cortex. The cotton wool plaques are similarly rounded structures as Arctic plaques, but the different antibodies stain them homogeneously, although with different intensities. The strong staining with abAβ_{x-42} (**a**) suggests that majority of Aβ terminates at aa 42, whereas Aβ_{x-40} (**b**) species are scarce. N-termini appear to be markedly variable including considerable amounts of N-terminally truncated Aβ species starting with pyroglutamate (Aβ_{11pE} or Aβ_{3pE}; **g** and **h**). The homogeneous staining suggests that the variably truncated Aβ species are relatively evenly distributed within the plaques. **i** and **j:** Sections from temporal cortex show accumulation of hp-tau positive NTs within Aβ plaques (six plaques marked with numbers). Note the NTs (arrows) also in the subpial Aβ–positive edging (asterisks) (*bar* in **a** 100 μm for **a-h**; *bar* in **i** 150 μm for **i** and **j**)
