# Supplementary material for: The Arctic AβPP mutation leads to Alzheimer’s disease pathology with highly variable topographic deposition of differentially truncated Aβ
Source: Acta Neuropathol Commun. 2013 Sep 10;1:60. doi: 10.1186/2051-5960-1-60 (PMC4226306; doi:10.1186/2051-5960-1-60)
Supplement: Additional file 2: Figure S7 — a: Sw2 patient’s striate area (visual cortex). The marked density of hp-tau positive NTs, including moderate density in layer 5, qualifies for the Braak stage VI of BrainNet Europe recommendation. The distribution of NTs by and large corresponds to that of Aβ plaques in the consecutive section c (asterisks). Note also the numerous Aβ-positive capillaries in layers 4–5. LFB-CV = Luxol fast blue-cresyl violet. (See also Figure 2d) (bar in a 400 μm for all panels). [file 2051-5960-1-60-S2.pdf]

Title: The *Arctic APP* mutation leads to Alzheimer's disease pathology with highly variable topographic deposition of differentially truncated Aβ

Journal: Acta Neuropathologica Communications

Authors: Hannu Kalimo[1], Maciej Lalowski, Nenad Bogdanovic, Ola Philipson, Thomas D. Bird , David Nochlin, Gerard D. Schellenberg, RoseMarie Brundin, Tommie Olofsson, Marc Baumann, Oliver Wirths, Thomas A. Bayer, Lars N.G. Nilsson, Hans Basun, Lars Lannfelt, Martin Ingelsson

Corresponding author: [1]Hannu Kalimo, Department of Pathology, University and University Hospital of Helsinki, Helsinki, Finland,

E-mail: hannu.kalimo@helsinki.fi

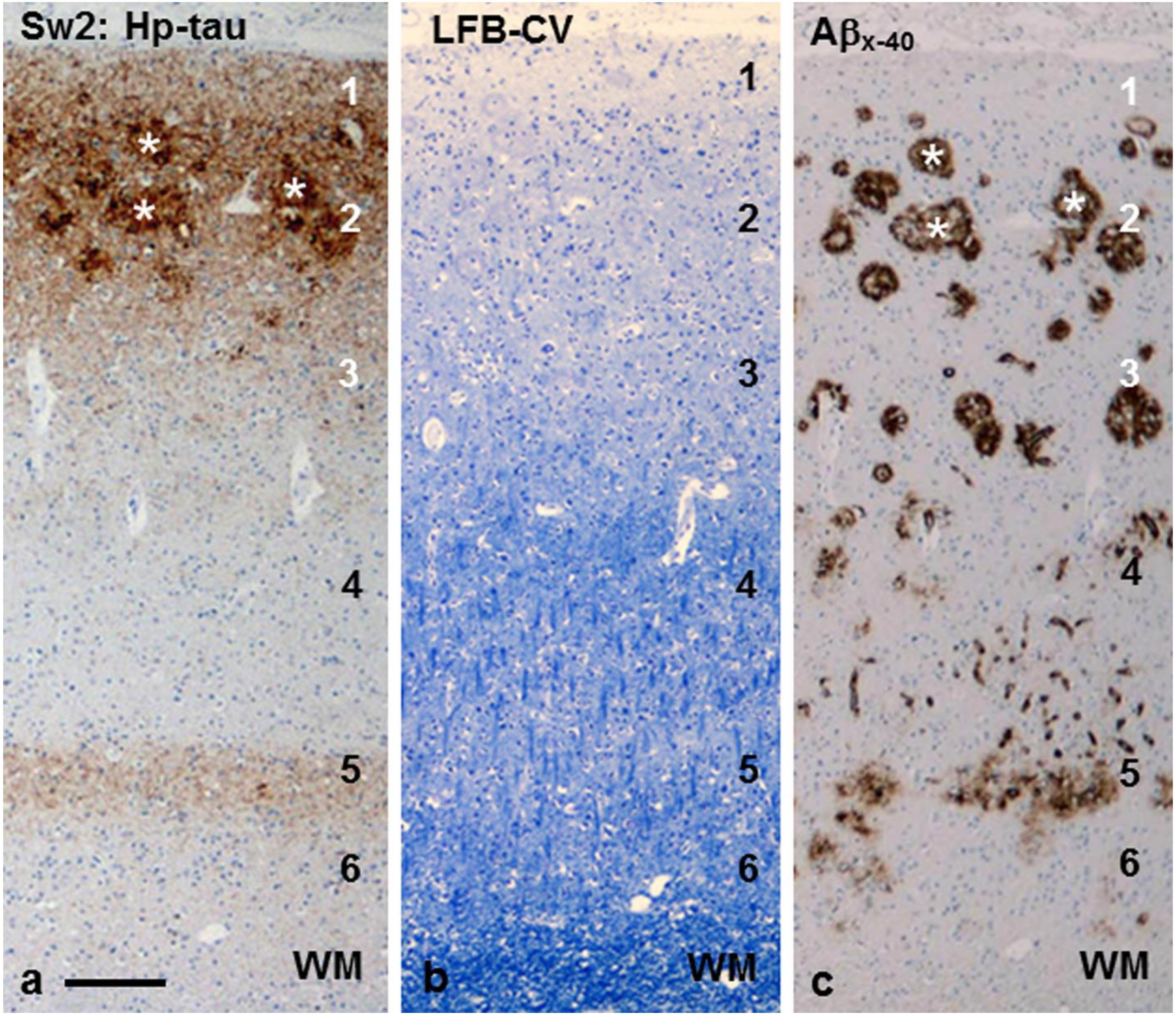

Sw2: Hp-tau
1
2
3
4
5
6
WM
a
LFB-CV
1
2
3
4
5
6
WM
b
$A\beta_{x-40}$
1
2
3
4
5
6
WM
c

Suppl. Fig. 7 a: Sw2 patient´s striate area (visual cortex). The marked density of hp-tau positive NTs, including moderate density in layer 5, qualifies for the Braak stage VI of BrainNet Europe recommendation. The distribution of NTs by and large corresponds to that of Aβ plaques in the consecutive section **c** (asterisks). Note also the numerous Aβ–positive capillaries in layers 4-5. LFB–CV = Luxol fast blue-cresyl violet. (See also Fig. 2d) (*bar* in **a** 400 μm for all panels)
