# Supplementary material for: The Arctic AβPP mutation leads to Alzheimer’s disease pathology with highly variable topographic deposition of differentially truncated Aβ
Source: Acta Neuropathol Commun. 2013 Sep 10;1:60. doi: 10.1186/2051-5960-1-60 (PMC4226306; doi:10.1186/2051-5960-1-60)
Supplement: Additional file 3: Figure S1 — In semiconsecutive sections from Am1 patient’s frontal cortex the plaques appear ring-shaped with C-terminal abAβx-42 and abAβx-40, mid-domain abAβ17-24 and Arctic specific abAβarc (a-c and g plus insets), whereas with more N-terminal (beyond aa 17) abAβ8-17, abAβ5-10 and abAβ1-5 (d-f) the plaques stain progressively weaker, although some plaques have intensely stained centres. Robust staining with abAβarc suggests an abundance of Arctic Aβ and a fair amount of Aβ appears to have pyroglutamate at positions 3 and 11 (h and i). The lesser number of plaques in layer 4 creates a sparsely populated band, best visible in b-d and g-i (similar band was observed in Am2 patient). No plaques are detectable in layer 1. Leptomeningeal blood vessels in b-i are strongly Aβ-positive, whereas in a they are only weakly positive. (bar in a 300 μm for all panels). [file 2051-5960-1-60-S3.pdf]

Title: The *Arctic APP* mutation leads to Alzheimer's disease pathology with highly variable topographic deposition of differentially truncated Aβ

Journal: Acta Neuropathologica Communications

Authors: Hannu Kalimo[1], Maciej Lalowski, Nenad Bogdanovic, Ola Philipson, Thomas D. Bird , David Nochlin, Gerard D. Schellenberg, RoseMarie Brundin, Tommie Olofsson, Marc Baumann, Oliver Wirths, Thomas A. Bayer, Lars N.G. Nilsson, Hans Basun, Lars Lannfelt, Martin Ingelsson

Corresponding author: [1]Hannu Kalimo, Department of Pathology, University and University Hospital of Helsinki, Helsinki, Finland,

E-mail: hannu.kalimo@helsinki.fi

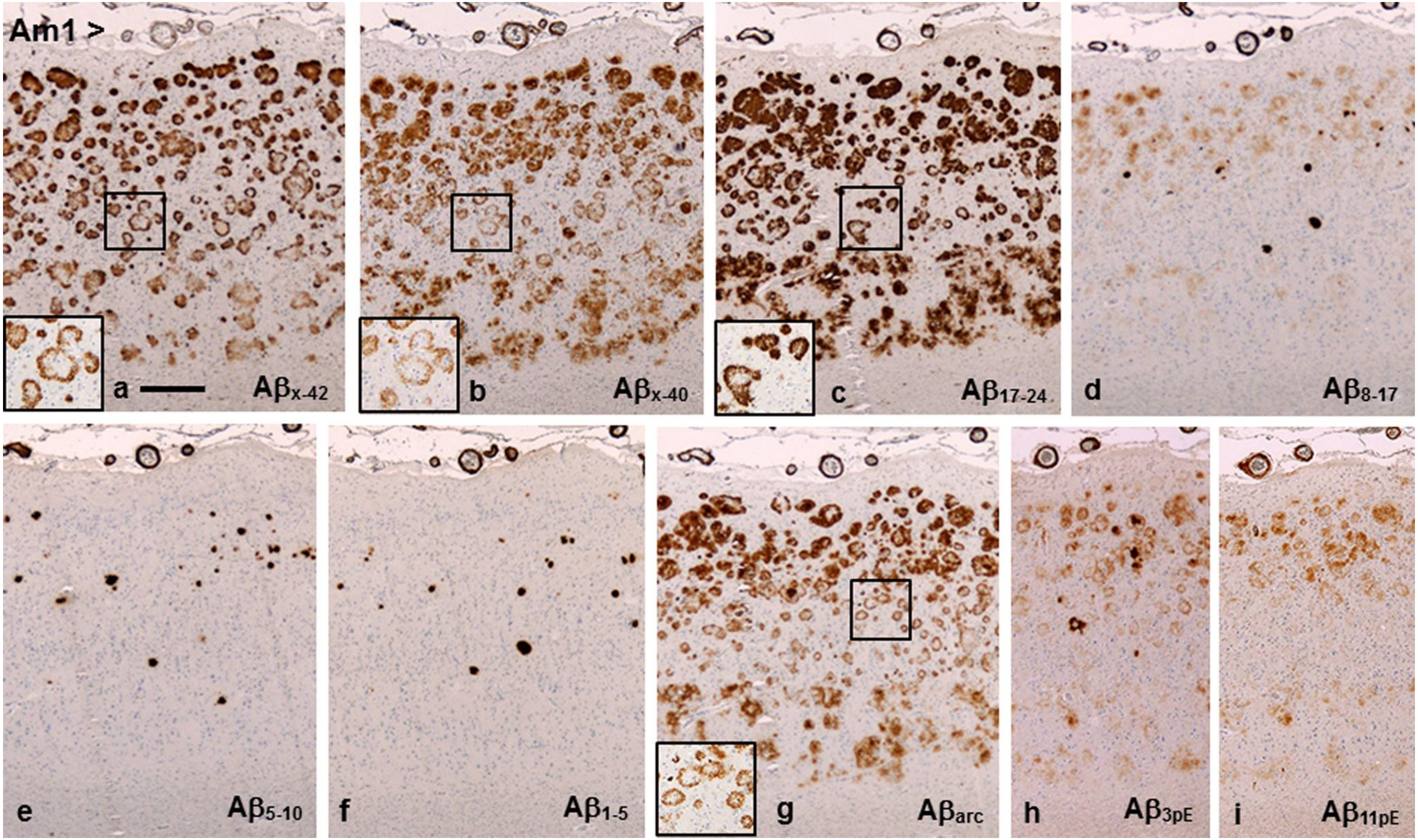

Am1 >
a
Aβx-42
b
Aβx-40
c
Aβ17-24
d
Aβ8-17
e
Aβ5-10
f
Aβ1-5
g
Aβarc
h
Aβ3pE
i
Aβ11pE

Suppl. Fig. 1 In semiconsecutive sections from Am1 patient´s frontal cortex the plaques appear ring-shaped with C-terminal abAβ_{x-42} and abAβ_{x-40}, mid-domain abAβ_{17-24} and Arctic specific abAβ_{arc} **(a-c** and **g** plus insets**)**, whereas with more N-terminal (beyond aa 17) abAβ_{8-17}, abAβ_{5-10} and abAβ_{1-5} (**d-f**) the plaques stain progressively weaker, although some plaques have intensely stained centres. Robust staining with abAβ_{arc} suggests an abundance of Arctic Aβ and a fair amount of Aβ appears to have pyroglutamate at positions 3 and 11 (**h** and **i**)**.** The lesser number of plaques in layer 4 creates a sparsely populated band, best visible in **b-d** and **g-i** (similar band was observed in Am2 patient). No plaques are detectable in layer 1. Leptomeningeal blood vessels in **b-i** are strongly Aβ-positive, whereas in **a** they are only weakly positive. (*bar* in **a** 300 µm for all panels)
