# Supplementary figures and images for: The Arctic AβPP mutation leads to Alzheimer’s disease pathology with highly variable topographic deposition of differentially truncated Aβ
Source: Acta Neuropathol Commun. 2013 Sep 10;1:60. doi: 10.1186/2051-5960-1-60 (PMC4226306; doi:10.1186/2051-5960-1-60)

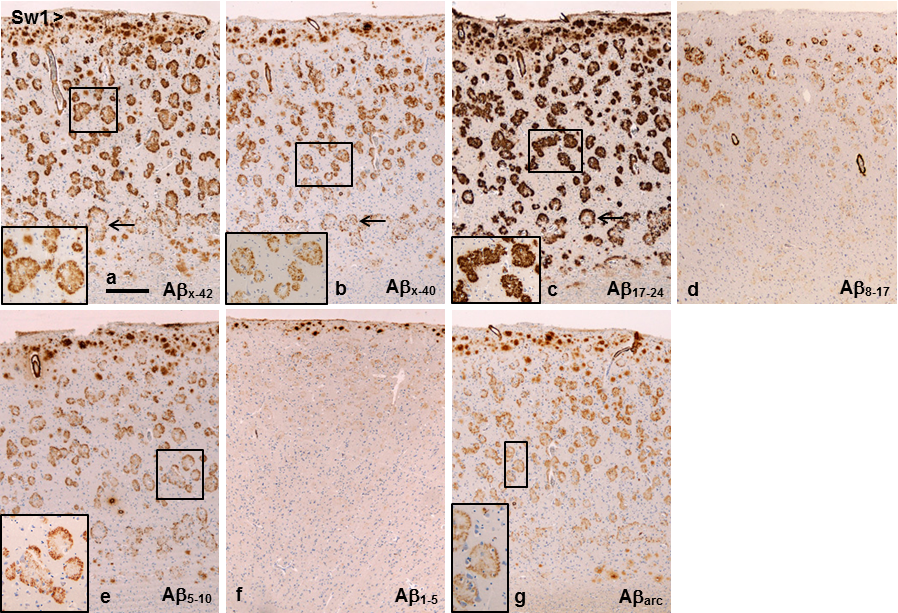

Supplement: Additional file 4: Figure S2 — Semiconsecutive sections from Sw1 patient’s frontal cortex. All antibodies except for abAβ1-5 disclose ring-shaped plaques (insets and arrows) in layers 2–6. The small subpial plaques in layer 1 are of diffuse type. Contrary to findings in patients Sw2 (Figure 4) and Am1 (Additional file 3: Figure S1) plaque centres are not intensely stained (bar in a 300 μm for all panels). [file 2051-5960-1-60-S4.tif]
