# Supplementary material for: The Arctic AβPP mutation leads to Alzheimer’s disease pathology with highly variable topographic deposition of differentially truncated Aβ
Source: Acta Neuropathol Commun. 2013 Sep 10;1:60. doi: 10.1186/2051-5960-1-60 (PMC4226306; doi:10.1186/2051-5960-1-60)
Supplement: Additional file 5: Table S1 — Aβ peptides detected in MALDI-TOF analyzes of immunoprecipitates from temporal cortex of Arctic AD Sw2 patient brain. Legend: Aβ peptides were measured in positive linear mode on AutoflexIII imager Bruker MALDI-TOF spectrometer in two independent experiments (named as exp1 and exp2). All m/z listed represent [Mav+H]+ (± 3 Da), after smoothing and background subtraction. Extraction of Aβ peptides was performed in 99% formic acid. Immunoprecipitation (IP) was performed with abAβ17-24 (Mab4G8) and abAβarc (Mab27) antibodies. Asterisks mark peaks labeled in Figure 3e. Molecular weight (m/z) in Daltons (Da). Relative intensities of the peaks in relation to the most intensive peak (Aβ17-43wt) were calculated with FlexAnalysis 3.4 software (Bruker Daltonics). The data from experiment 1 were presented in Philipson et al. Neurobiol Aging (2012), 33: 1010 e1011-1013 [18]. [file 2051-5960-1-60-S5.pdf]

Supplemental Table 1. Aβ peptides detected in MALDI-TOF analyzes of immunoprecipitates from temporal cortex of Arctic AD Sw2 patient brain.

| Aβ peptide sequence | Aβ m/z predicted | IP: abA$\beta_{17-24}$/abA$\beta_{arc}$ m/z_exp1 | IP: abA$\beta_{17-24}$/abA$\beta_{arc}$ m/z_exp2 | IP: abA$\beta_{17-24}$/abA$\beta_{arc}$ Relative Intensity_exp1 | IP: abA$\beta_{17-24}$/abA$\beta_{arc}$ Relative Intensity_exp2 |
|---|---|---|---|---|---|
| 18-42wt | 2464.9 | 2463,471 | 2463,751 | 0,156 | 0,151 |
| 17-43wt | 2679.2 | 2678,310 | 2678,711 | 1,000 | 1,000 |
| 11pE-40arc* | 3062.6 | 3062,808 | 3061,445 | 0,222 | 0,200 |
| 11pE-42arc* | 3246.8 | 3246,181 | 3245,964 | 0,335 | 0,295 |
| 9-42arc | 3485.1 | 3482,913 | 3484,111 | 0,460 | 0,450 |
| 7-40arc formylated | 3531.0 | 3530,302 | 3530,275 | 0,682 | 0,570 |
| 10-42wt formylated | 3528.0 | 3530,302 | 3530,275 | 0,682 | 0,570 |
| 6-40wt oxidized | 3728.2 | 3728,588 | 3728,212 | 0,109 | 0,114 |
| 5-40arc | 3796.3 | 3792,777 | 3792,398 | 0,147 | 0,132 |
| 5-40wt oxidized | 3884.4 | 3886,251 | 3886,635 | 0,241 | 0,198 |
| 4-40arc | 3943.5 | 3943,952 | 3944,181 | 0,438 | 0,409 |
| 5-42arc oxidized | 3996.6 | 3997,343 | 3997,012 | 0,322 | 0,280 |
| 3pE-40arc* | 4054.6 | 4056,985 | 4056,985 | 0,163 | 0,204 |
| 1-40arc* | 4258.8 | 4259,081 | 4258,680 | 0,140 | 0,123 |
| 1-42arc* | 4443.1 | 4442,975 | 4443,544 | 0,438 | 0,375 |
| 1-45wt | 4828.5 | 4828,407 | 4828,767 | 0,276 | 0,255 |

**Legend:** Aβ peptides were measured in positive linear mode on AutoflexIII imager Bruker MALDI-TOF spectrometer in two independent experiments (named as exp1 and exp2). All *m/z* listed represent $[M_{av}+H]^+$ (± 3 Da), after smoothing and background subtraction. Extraction of Aβ peptides was performed in 99% formic acid. Immunoprecipitation (IP) was performed with abA$\beta_{17-24}$ (Mab4G8) and abA$\beta_{arc}$ (Mab27) antibodies. Asterisks mark peaks labeled in **Figure 3e**. Molecular weight (m/z) in Daltons (Da). Relative intensities of the peaks in relation to the most intensive peak (Aβ17-43wt) were calculated with FlexAnalysis 3.4 software (Bruker Daltonics). The data from experiment 1 were presented in Philipson et al. *Neurobiol Aging* (2012), **33**: 1010 e1011-1013 [35].
