# Supplementary material for: The Arctic AβPP mutation leads to Alzheimer’s disease pathology with highly variable topographic deposition of differentially truncated Aβ
Source: Acta Neuropathol Commun. 2013 Sep 10;1:60. doi: 10.1186/2051-5960-1-60 (PMC4226306; doi:10.1186/2051-5960-1-60)
Supplement: Additional file 6: Figure S3 — Sw2 patient’s hippocampus. a: In H&E stained sections the plaques are difficult to discern, except when they are located within the dentate gyrus displacing granule cells (arrows). b: With Bielschowsky silver impregnation DNs within plaques are clearly visible, whereas plaques themselves are inconspicuous. Inset: NFT in a hippocampal pyramidal neuron is strongly silver positive. (bar in a 100 μm for a and b). [file 2051-5960-1-60-S6.pdf]

Title: The *Arctic APP* mutation leads to Alzheimer's disease pathology with highly variable topographic deposition of differentially truncated Aβ

Journal: Acta Neuropathologica Communications

Authors: Hannu Kalimo[1], Maciej Lalowski, Nenad Bogdanovic, Ola Philipson, Thomas D. Bird , David Nochlin, Gerard D. Schellenberg, RoseMarie Brundin, Tommie Olofsson, Marc Baumann, Oliver Wirths, Thomas A. Bayer, Lars N.G. Nilsson, Hans Basun, Lars Lannfelt, Martin Ingelsson

Corresponding author: [1]Hannu Kalimo, Department of Pathology, University and University Hospital of Helsinki, Helsinki, Finland,

E-mail: hannu.kalimo@helsinki.fi

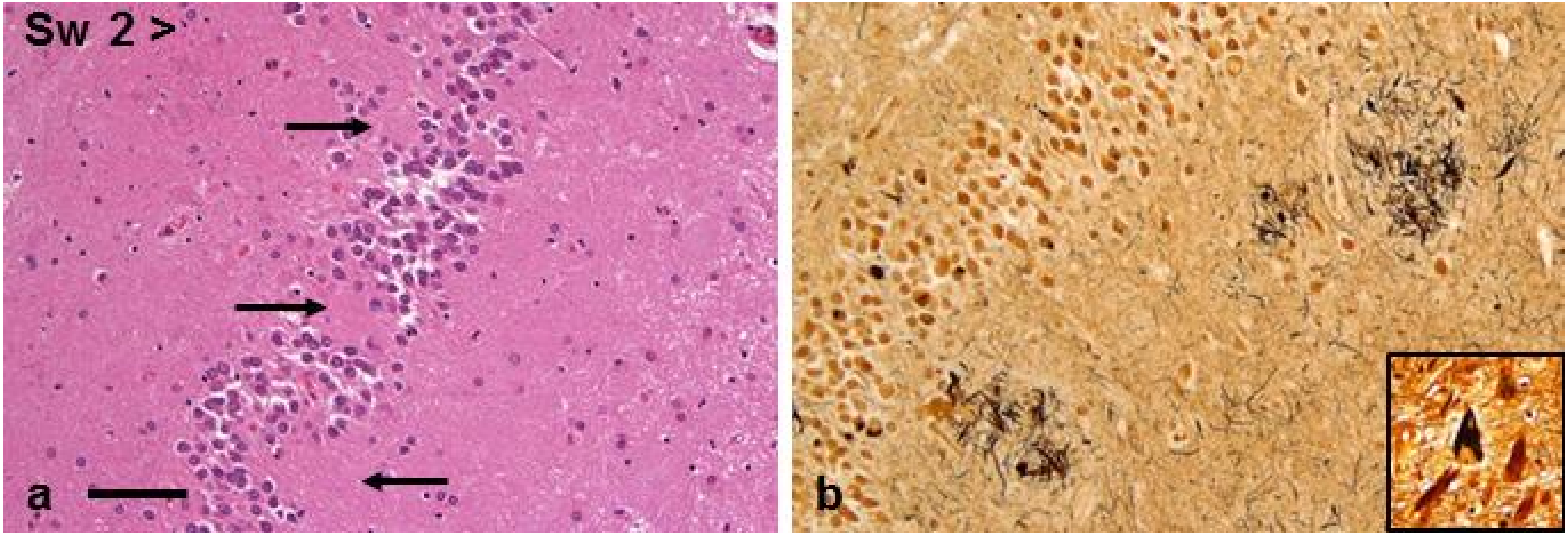
Sw 2 >
a
b

Suppl. Fig. 3 Sw2 patient´s hippocampus. **a:** In H&E stained sections the plaques are difficult to discern, except when they are located within the dentate gyrus displacing granule cells (arrows). **b:** With Bielschowsky silver impregnation DNs within plaques are clearly visible, whereas plaques themselves are inconspicuous. **Inset:** NFT in a hippocampal pyramidal neuron is strongly silver positive. (*bar* in **a** 100 μm for **a** and **b**)
