# Supplementary material for: The Arctic AβPP mutation leads to Alzheimer’s disease pathology with highly variable topographic deposition of differentially truncated Aβ
Source: Acta Neuropathol Commun. 2013 Sep 10;1:60. doi: 10.1186/2051-5960-1-60 (PMC4226306; doi:10.1186/2051-5960-1-60)
Supplement: Additional file 7: Figure S4 — a-g: Aβ-plaques in Swe2 patient’s claustrum show similar targetoid pattern as in neocortex (a-c consecutive sections). a: With abAβx-42 dark corona and pale centre. b: With abAβx-40 fair staining of both centre and corona. c: With abAβ1-5 dark centre and pale corona. d: Middomain abAβ17-24 stains strongly both centre and corona. e: Specific abAβarc. gives similar pattern as abAβ17-24, though with much lesser intensity. f and g: Plaques comprise of both Aβ3pE and Aβ11pE, though less of the latter. h-k: Plaques in Sw2 patient’s putamen are small and diffusely stained. The most intense stainings are seen with abAβx-42, abAβarc and abAβ3pE (h, j and k) suggesting an abundance of Aβ with pyroglutamate-modified N-termini, which is consistent with the virtually negative abAβ1-5 staining (i). l: In Sw2 patient’s amygdala plaques are similar as in putamen but more numerous. m: In Sw2 patient’s thalamus the plaques are ragged and weakly stained. (bar in a 100 μm for a-c; bar in d 100 μm for d-g; bar in h 50 μm for h-l; bar in m 50 μm). [file 2051-5960-1-60-S7.pdf]

Title: The *Arctic APP* mutation leads to Alzheimer's disease pathology with highly variable topographic deposition of differentially truncated Aβ

Journal: Acta Neuropathologica Communications

Authors: Hannu Kalimo[1], Maciej Lalowski, Nenad Bogdanovic, Ola Philipson, Thomas D. Bird , David Nochlin, Gerard D. Schellenberg, RoseMarie Brundin, Tommie Olofsson, Marc Baumann, Oliver Wirths, Thomas A. Bayer, Lars N.G. Nilsson, Hans Basun, Lars Lannfelt, Martin Ingelsson

Corresponding author: [1]Hannu Kalimo, Department of Pathology, University and University Hospital of Helsinki, Helsinki, Finland,

E-mail: hannu.kalimo@helsinki.fi

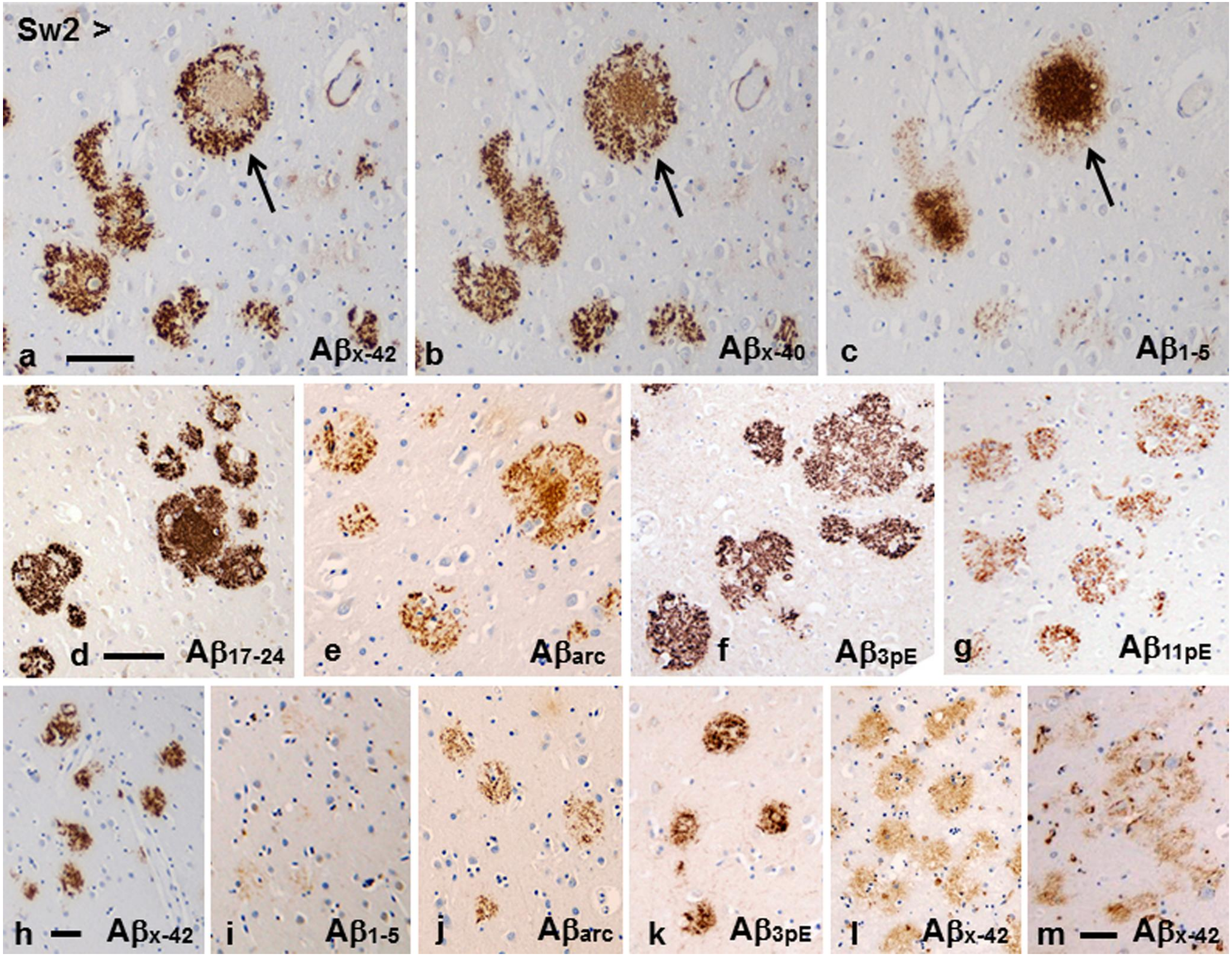
Sw2 >
a
Aβx-42
b
Aβx-40
c
Aβ1-5
d
Aβ17-24
e
Aβarc
f
Aβ3pE
g
Aβ11pE
h
Aβx-42
i
Aβ1-5
j
Aβarc
k
Aβ3pE
l
Aβx-42
m
Aβx-42

Suppl. Fig. 4 a-g: Aβ-plaques in Swe2 patient´s claustrum show similar targetoid pattern as in neocortex (**a-c** consecutive sections). **a:** With abAβ$_{x-42}$ dark corona and pale centre**. b:** With abAβ$_{x-40}$ fair staining of both centre and corona. **c:** With abAβ$_{1-5}$ dark centre and pale corona. **d:** Mid-domain abAβ$_{17-24}$ stains strongly both centre and corona. **e:** Specific abAβ$_{arc}$. gives similar pattern as abAβ$_{17-24}$, though with much lesser intensity. **f** and **g:** Plaques comprise of both Aβ$_{3pE}$ and Aβ$_{11pE}$, though less of the latter. **h-k:** Plaques in Sw2 patient´s putamen are small and diffusely stained**.** The most intense stainings are seen with abAβ$_{x-42}$, abAβ$_{arc}$ and abAβ$_{3pE}$ (**h, j** and **k**) suggesting an abundance of Aβ with pyroglutamate-modified N-termini, which is consistent with the virtually negative abAβ$_{1-5}$ staining (**i**). **l**: In Sw2 patient´s amygdala plaques are similar as in putamen but more numerous. **m:** In Sw2 patient´s thalamus the plaques are ragged and weakly stained. (*bar* in **a** 100 µm for **a-c**; *bar* in **d** 100 µm for **d-g**; *bar* in **h** 50 µm for **h-l**; *bar* in **m** 50 µm)
