# Supplementary material for: The Arctic AβPP mutation leads to Alzheimer’s disease pathology with highly variable topographic deposition of differentially truncated Aβ
Source: Acta Neuropathol Commun. 2013 Sep 10;1:60. doi: 10.1186/2051-5960-1-60 (PMC4226306; doi:10.1186/2051-5960-1-60)
Supplement: Additional file 8: Figure S5 — a-k: Sw2 patient’s medulla. A few compact plaques are positive with C-terminal (a), mid-domain (b) and N-terminal antibodies (c). Remarkably, abAβx-42 renders the neuropil in inferior olivary nucleus distinctly positive (a and d), whereas with the other antibodies it is negative (b, c and e-i). Both abAβx-42 and abAβ17-24, (d and e; arrows), but not the rest of Aβ antibodies applied (f-i), stain granular inclusions in the cytoplasm of seemingly well preserved olivary neurons within and adjacent to plaques. The neuronal inclusions also stain with PAS (j) and an antibody to lysosomal cathepsin D (k). (bar in a 350 μm for a-c; bar in d 50 μm for d-i; bar in j 25 μm; bar in k 30 μm). [file 2051-5960-1-60-S8.pdf]

Title: The *Arctic APP* mutation leads to Alzheimer's disease pathology with highly variable topographic deposition of differentially truncated Aβ

Journal: Acta Neuropathologica Communications

Authors: Hannu Kalimo[1], Maciej Lalowski, Nenad Bogdanovic, Ola Philipson, Thomas D. Bird , David Nochlin, Gerard D. Schellenberg, RoseMarie Brundin, Tommie Olofsson, Marc Baumann, Oliver Wirths, Thomas A. Bayer, Lars N.G. Nilsson, Hans Basun, Lars Lannfelt, Martin Ingelsson

Corresponding author: [1]Hannu Kalimo, Department of Pathology, University and University Hospital of Helsinki, Helsinki, Finland,

E-mail: hannu.kalimo@helsinki.fi

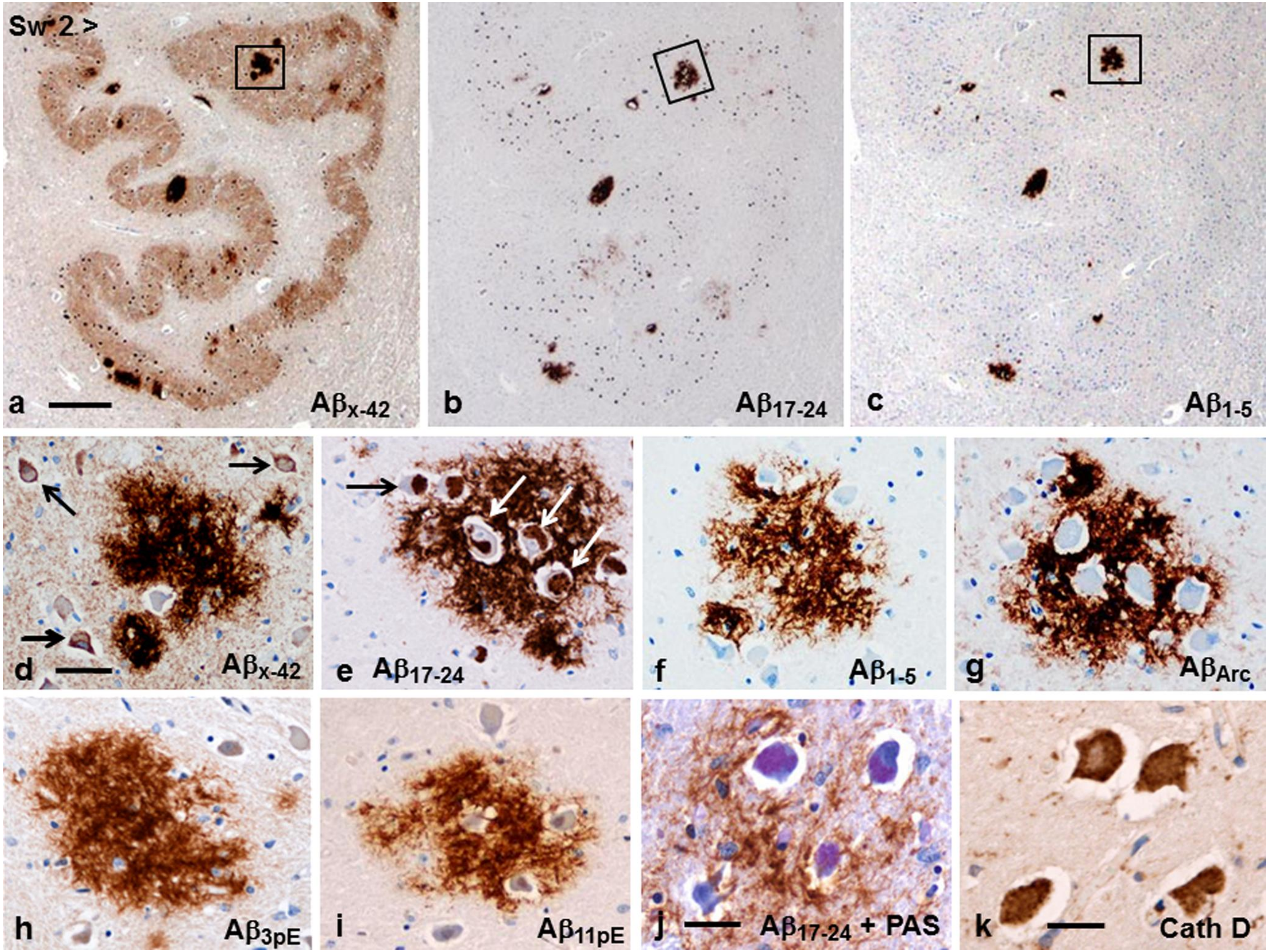

Sw 2 >
a
Aβx-42
b
Aβ17-24
c
Aβ1-5
d
Aβx-42
e
Aβ17-24
f
Aβ1-5
g
AβArc
h
Aβ3pE
i
Aβ11pE
j
Aβ17-24 + PAS
k
Cath D

Suppl. Fig. 5 a-k: Sw2 patient´s medulla. A few compact plaques are positive with C-terminal **(a)**, mid-domain **(b)** and N-terminal antibodies **(c)**. Remarkably, abA$\beta_{x\text{-}42}$ renders the neuropil in inferior olivary nucleus distinctly positive **(a** and **d)**, whereas with the other antibodies it is negative **(b, c** and **e-i)**. Both abA$\beta_{x\text{-}42}$ and abA$\beta_{17\text{-}24}$, **(d** and **e**; arrows**)**, but not the rest of Aβ antibodies applied **(f-i)**, stain granular inclusions in the cytoplasm of seemingly well preserved olivary neurons within and adjacent to plaques**.** The neuronal inclusions also stain with PAS **(j)** and an antibody to lysosomal cathepsin D **(k)**. (*bar* in **a** 350 μm for **a-c**; *bar* in **d** 50 μm **for d-i**; *bar* in **j** 25 μm; *bar* in **k** 30 μm)
