# Supplementary material for: The Arctic AβPP mutation leads to Alzheimer’s disease pathology with highly variable topographic deposition of differentially truncated Aβ
Source: Acta Neuropathol Commun. 2013 Sep 10;1:60. doi: 10.1186/2051-5960-1-60 (PMC4226306; doi:10.1186/2051-5960-1-60)
Supplement: Additional file 9: Figure S6 — a-i: Immunostainings of Am1 patient’s cerebellum demonstrates the marked interindividual variation despite the same genetic defect (cf. Figure 5). Only abAβx-42 and abAβ17-24 are clearly positive (a and c), whereas abAβx-40 (b) and the more N-terminal abAβ8-17, abAβ5-10 and abAβ1-5 (d-f) give virtually no parenchymal staining, even though the blood vessels are strongly positive. Weak staining with abAβarc (g) is consistent with most parenchymal deposits being composed of wild-type Aβ. A fair proportion of the deposited Aβ appears to have pyroglutamate N-termini (h and i). (bar in a 150 μm for all panels). [file 2051-5960-1-60-S9.pdf]

Title: The *Arctic APP* mutation leads to Alzheimer's disease pathology with highly variable topographic deposition of differentially truncated Aβ

Journal: Acta Neuropathologica Communications

Authors: Hannu Kalimo[1], Maciej Lalowski, Nenad Bogdanovic, Ola Philipson, Thomas D. Bird , David Nochlin, Gerard D. Schellenberg, RoseMarie Brundin, Tommie Olofsson, Marc Baumann, Oliver Wirths, Thomas A. Bayer, Lars N.G. Nilsson, Hans Basun, Lars Lannfelt, Martin Ingelsson

Corresponding author: [1]Hannu Kalimo, Department of Pathology, University and University Hospital of Helsinki, Helsinki, Finland,

E-mail: hannu.kalimo@helsinki.fi

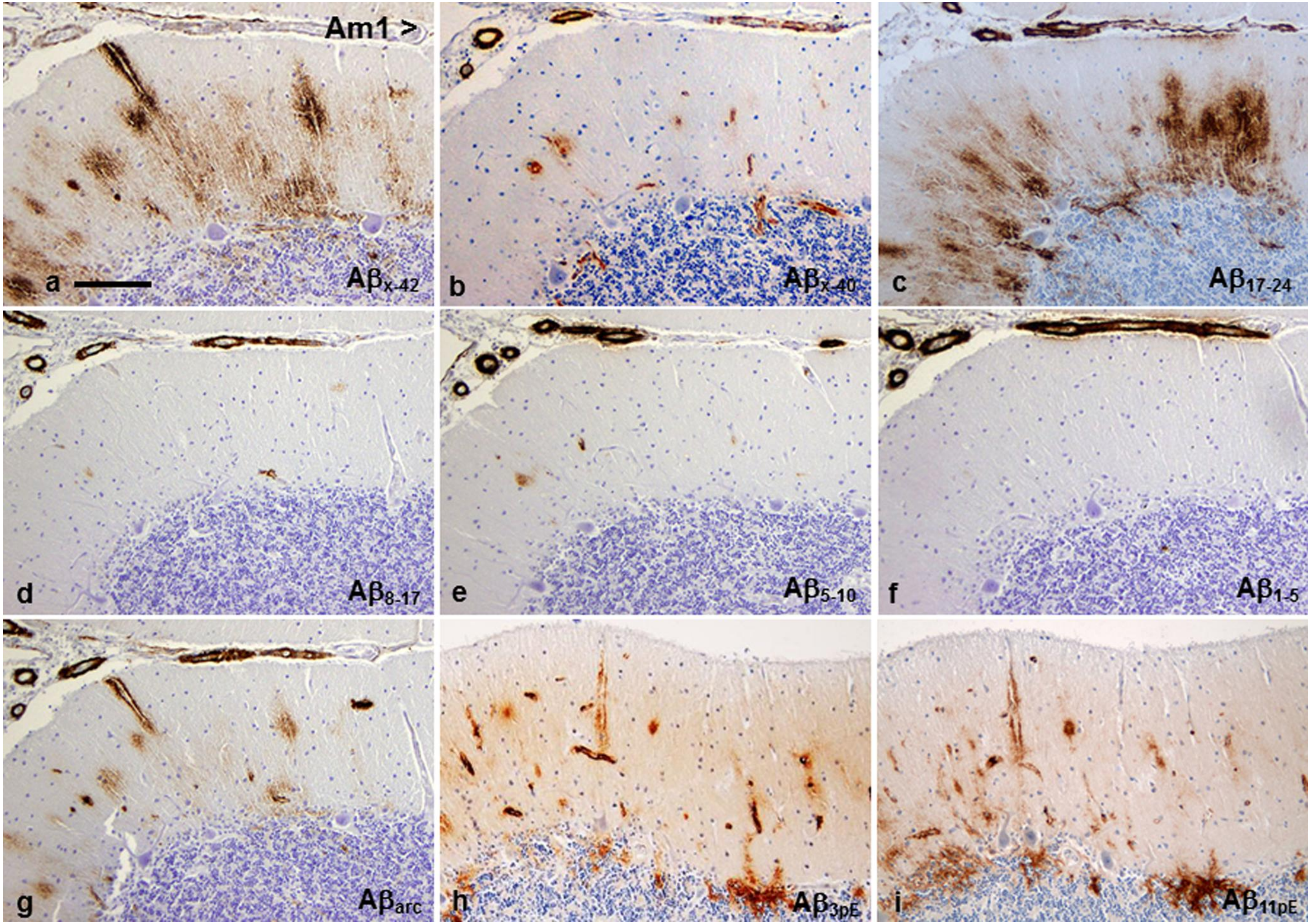
Am1 >
a
Aβx-42
b
Aβx-40
c
Aβ17-24
d
Aβ8-17
e
Aβ5-10
f
Aβ1-5
g
Aβarc
h
Aβ3pE
i
Aβ11pE

Suppl. Fig. 6 a-i: Immunostainings of Am1 patient´s cerebellum demonstrates the marked inter-individual variation despite the same genetic defect (cf. Fig. 5). Only abAβ_{x-42} and abAβ_{17-24} are clearly positive (**a** and **c**), whereas abAβ_{x-40} (**b**) and the more N-terminal abAβ_{8-17}, abAβ_{5-10} and abAβ_{1-5} (**d-f**) give virtually no parenchymal staining, even though the blood vessels are strongly positive. Weak staining with abAβ_{arc} (**g**) is consistent with most parenchymal deposits being composed of wild-type Aβ. A fair proportion of the deposited Aβ appears to have pyroglutamate N-termini (**h** and **i**). (*bar* in **a** 150 μm for all panels)
