# Supplementary material for: The Arctic AβPP mutation leads to Alzheimer’s disease pathology with highly variable topographic deposition of differentially truncated Aβ
Source: Acta Neuropathol Commun. 2013 Sep 10;1:60. doi: 10.1186/2051-5960-1-60 (PMC4226306; doi:10.1186/2051-5960-1-60)
Supplement: Additional file 10: Figure S8 — Sw2 patient’s cerebellum. a: The density of GFAP-positive network is prominent, especially in the molecular layer. b: The density of the network corresponds to the deposition of Aβ in the Purkinje cell layer, but it differs from the perivascular accentuation of Aβ in the molecular layer. c: The microglial reaction to Aβ is minimal (bar in a 200 μm for all panels). [file 2051-5960-1-60-S10.pdf]

Title: The *Arctic APP* mutation leads to Alzheimer's disease pathology with highly variable topographic deposition of differentially truncated Aβ

Journal: Acta Neuropathologica Communications

Authors: Hannu Kalimo[1], Maciej Lalowski, Nenad Bogdanovic, Ola Philipson, Thomas D. Bird , David Nochlin, Gerard D. Schellenberg, RoseMarie Brundin, Tommie Olofsson, Marc Baumann, Oliver Wirths, Thomas A. Bayer, Lars N.G. Nilsson, Hans Basun, Lars Lannfelt, Martin Ingelsson

Corresponding author: [1]Hannu Kalimo, Department of Pathology, University and University Hospital of Helsinki, Helsinki, Finland,

E-mail: hannu.kalimo@helsinki.fi

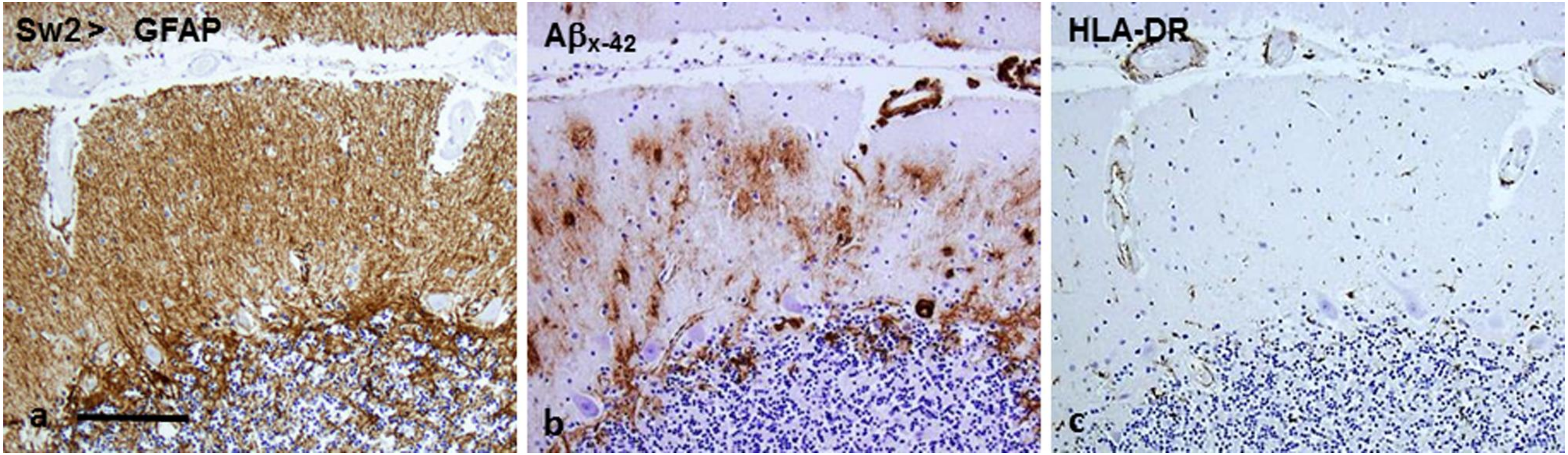
Sw2 > GFAP
a
Aβx-42
b
HLA-DR
c

Suppl. Fig. 8 Sw2 patient´s cerebellum. **a:** The density of GFAP-positive network is prominent, especially in the molecular layer. **b:** The density of the network corresponds to the deposition of Aβ in the Purkinje cell layer, but it differs from the perivascular perivascularaccentuation of Aβ in the molecular layer. **c:** The microglial reaction to Aβ is minimal (*bar* in **a** 200 μm for all panels)
